# Fast and flexible bacterial genomic epidemiology with PopPUNK

**DOI:** 10.1101/gr.241455.118

**Published:** 2019-02

**Authors:** John A. Lees, Simon R. Harris, Gerry Tonkin-Hill, Rebecca A. Gladstone, Stephanie W. Lo, Jeffrey N. Weiser, Jukka Corander, Stephen D. Bentley, Nicholas J. Croucher

**Affiliations:** 1 Department of Microbiology, New York University School of Medicine, New York, New York 10016, USA;; 2 Parasites and Microbes, Wellcome Sanger Institute, Wellcome Genome Campus, Hinxton CB10 1SA, United Kingdom;; 3 Department of Biostatistics, University of Oslo, 0372 Oslo, Norway;; 4 Helsinki Institute of Information Technology, Department of Mathematics and Statistics, University of Helsinki, 00014 Helsinki, Finland;; 5 Institute of Infection and Global Health, University of Liverpool, Liverpool L7 3EA, United Kingdom;; 6 Department of Pathology, University of Cambridge, Cambridge CB2 1QP, United Kingdom;; 7 MRC Centre for Global Infectious Disease Analysis, Department of Infectious Disease Epidemiology, Imperial College London, London W2 1PG, United Kingdom

## Abstract

The routine use of genomics for disease surveillance provides the opportunity for high-resolution bacterial epidemiology. Current whole-genome clustering and multilocus typing approaches do not fully exploit core and accessory genomic variation, and they cannot both automatically identify, and subsequently expand, clusters of significantly similar isolates in large data sets spanning entire species. Here, we describe PopPUNK (Population Partitioning Using Nucleotide *K*-mers), a software implementing scalable and expandable annotation- and alignment-free methods for population analysis and clustering. Variable-length *k*-mer comparisons are used to distinguish isolates’ divergence in shared sequence and gene content, which we demonstrate to be accurate over multiple orders of magnitude using data from both simulations and genomic collections representing 10 taxonomically widespread species. Connections between closely related isolates of the same strain are robustly identified, despite interspecies variation in the pairwise distance distributions that reflects species’ diverse evolutionary patterns. PopPUNK can process 10^3^–10^4^ genomes in a single batch, with minimal memory use and runtimes up to 200-fold faster than existing model-based methods. Clusters of strains remain consistent as new batches of genomes are added, which is achieved without needing to reanalyze all genomes de novo. This facilitates real-time surveillance with consistent cluster naming between studies and allows for outbreak detection using hundreds of genomes in minutes. Interactive visualization and online publication is streamlined through the automatic output of results to multiple platforms. PopPUNK has been designed as a flexible platform that addresses important issues with currently used whole-genome clustering and typing methods, and has potential uses across bacterial genetics and public health research.

Determining whether a set of pathogen isolates are significantly more genetically similar than randomly selected representatives from the circulating population is critical in identifying transmission pairs, localized outbreaks, or global patterns of dissemination (Croucher and Didelot 2015). For phenotypically diverse bacterial pathogens, categorizing sets of similar isolates is particularly valuable, as such clusters of genotypes are often strongly associated with variation in clinically relevant traits, including host range (Willems et al. 2012; Reuter et al. 2014; Weinert et al. 2015), virulence (Reuter et al. 2014; Weinert et al. 2015; Alikhan et al. 2018), propensity to cause nosocomial outbreaks (Willems et al. 2012; Aanensen et al. 2016), and antimicrobial resistance profile (Aanensen et al. 2016; Kallonen et al. 2017). These subdivisions are also of practical importance for phylodynamic studies (Croucher et al. 2013; Weinert et al. 2015; Kallonen et al. 2017; Kremer et al. 2017) or recombination identification (Croucher et al. 2013; Weinert et al. 2015). Cluster identification has typically used complex population structure analysis models, such as hierBAPS (Cheng et al. 2013), but these computationally intensive methods are not optimal for ongoing surveillance, as they must be rerun from scratch when data sets expand, and low-frequency clusters tend to be merged into a single diverse group (Croucher et al. 2013; Grad et al. 2016; Willemse et al. 2016). This is important, as following the temporal trends in these clusters provides critical information on population-level changes following the emergence of new genotypes (Kallonen et al. 2017) or resulting from interventions such as vaccine introduction (Croucher et al. 2013). Therefore devising efficient, extendable population structure analysis algorithms represents a critical challenge as genomic pathogen surveillance becomes routine.

Existing approaches to using genetic data for surveillance are typically based on multilocus sequence typing (MLST), in which isolates are labeled according to their set of alleles at several short fragments of unlinked housekeeping genes. Following its inception in the late 1990s (Maiden et al. 1998), continually updated online MLST databases have facilitated rapid comparisons between global isolate sets collected over decades (Aanensen and Spratt 2005; Jolley et al. 2017). Clusters within the population can be defined through grouping similar MLST sequence types using minimum-spanning trees, such as those produced by eBURST (Feil et al. 2004). However, the fixed resolution of MLST means it struggles to distinguish isolates of low-diversity pathogens such as *Mycobacterium tuberculosis* and *Salmonella enterica* serovar Typhi (Achtman 2012). Similarly, distinct clusters of high-diversity pathogens can be merged into “straggly” clonal complexes encompassing highly divergent bacteria, owing to high recombination rates causing spurious links between unrelated groups of isolates (Turner et al. 2007; Willems et al. 2012). Whole-genome sequence data provide an opportunity to greatly improve the precision and resolution of bacterial typing. Core genome MLST (cgMLST) schemes extend the MLST approach across sequences common to all isolates of a sample and have demonstrated their value at scales ranging from genus-wide taxonomy to investigation of nosocomial outbreaks in *Neisseria* spp. (Bratcher et al. 2014), *Listeria monocytogenes* (Ruppitsch et al. 2015), *Enterococcus faecium* (De Been et al. 2015), *Escherichia coli*, *Pseudomonas aeruginosa*, *Klebsiella pneumoniae*, and *Staphylococcus aureus* (Mellmann et al. 2016). This combines the speed and ease of assigning indices to alleles combined with the increased resolution of using larger proportions of the core genome. However, all such analyses are limited to the coding sequences identified in the original scheme: In the species-specific schemes listed above, this varied between 41% and 84% of the genes in a typical genome, which can be further limited if not all loci can be extracted from the query genome. Further resolution is lost if these data are treated as a set of allele identifiers, rather than nucleotide sequences, as this obscures the level of similarity between nonidentical alleles. Nevertheless, cgMLST is highly sensitive and can uncover deeper relationships between isolates than MLST. This is a trade-off with specificity, since a minimum-spanning tree constructed using sequence types is fully connected, losing the simple and intuitive splitting of the population into clonal complexes (Feil et al. 2004).

To improve on the resolution of cgMLST, whole-genome MLST (wgMLST) schemes have been developed to incorporate accessory genes. Differences in gene content underlie much phenotypic variation between bacteria, and these are correlated with core genome divergence in multiple species (Croucher et al. 2014; Zhou et al. 2014; Holt et al. 2015; Aanensen et al. 2016), motivating the use of this information in epidemiological typing. However, incorporating data from accessory loci can be difficult, as many are more difficult to align and define than core genes. Further complications arise from the difficulty of resolving orthologous and paralogous genes (Zhang et al. 2015) and the capacity of mobile elements to import many genes over short timescales (Croucher et al. 2016; Abudahab et al. 2018), potentially confounding outbreak identification (Zhou et al. 2013). Some wgMLST schemes add further complexity through continually expanding the scheme to incorporate new loci. This necessitates extracting previously unseen loci from query genomes and ensuring consistency across the revised scheme, which is both computationally intensive and algorithmically complicated. Instead, wgMLST schemes may be fixed, although this limits their resolution by excluding newly observed accessory genes. These will include any newly emerged loci that enter the population through horizontal gene transfer (Alikhan et al. 2018), the detection of which represents a critical aspect of pathogen surveillance.

The population structures identified by cgMLST (Maiden and Harrison 2016; Henri et al. 2017; Alikhan et al. 2018), wgMLST (Henri et al. 2017; Alikhan et al. 2018), and hierBAPS (Croucher et al. 2013; Aanensen et al. 2016; Kallonen et al. 2017; Alikhan et al. 2018) are highly consistent with the MLST clonal complexes, indicating that where such clusters can be identified, they are biologically meaningful. However, none of the existing genomic approaches are appropriate for defining these clusters in an easily extendable manner from genomic surveillance data. To solve these methodological difficulties in a single approach, we have developed PopPUNK (Population Partitioning Using Nucleotide *K*-mers; https://poppunk.readthedocs.io/), which uses variable-length *k*-mer comparisons to find genetic distances between isolates, the distribution of which is then used to find clusters we define as strains, sets of isolates significantly similar in both their core and accessory genomes relative to the rest of the species.

## Results

### PopPUNK uses variable-length *k*-mers to accurately resolve genetic divergence

We proposed the probability that a *k*-mer will match between a pair of sequences, *p*_match_, is the product of *p*_accessory_, the probability it does not represent an accessory locus unique to one member of the pair, and *p*_core_, the probability it represents a shared core genome sequence that does not contain any mismatches. To calculate *p*_core_ and *p*_accessory_, comparisons were run using the MinHash algorithm (Broder 1997) as implemented in Mash (Ondov et al. 2016), which estimates the Jaccard similarity between reduced size *k*-mer “sketches” of the two sequences, providing a measure of *p*_match_. This is run for a selection of *k*-mer lengths between *k*_min_ and *k*_max_, the former being determined by the minimum sequence length needed to avoid frequent false positive matches given the size of the genomes being compared (Methods), and the latter is limited by the requirement for memory-efficient MinHash processing. By determining the probability of *k*-mer matching shared core sequence over this size range, it is possible to estimate the density of single-nucleotide polymorphisms (SNPs) distinguishing the pair across their shared core, defined as π (Nei and Li 1979). This is because longer *k*-mers are more likely to contain a SNP, and correspondingly the probability of a *k*-mer perfectly matching between a pair of isolates decreases by a factor of (1 − π) for each additional base in the *k*-mer:

$$p_{\mathrm{core}}={\left(1-\pi \right)}^{k}.$$

This approach assumes a random and uniform distribution of SNPs across the core, which is defined as those genomic regions in which nucleotide strings at least *k*_min_ long can be matched, representing a significant level of similarity between the pair. Loci in which there are no *k*_min_-long matches, resulting from either absence of the sequence in one member of the pair, or high sequence divergence of at least one SNP per *k*_min_ bases, are classified as belonging to the accessory genome; *k*_min_ thereby provides an intrinsic statistical distinction between the core and accessory regions in the pairwise comparison. Hence, *p*_accessory_ can be regarded as the Jaccard similarity between a pair in terms of the extent of common sequence they share, allowing the definition of the accessory divergence between sequences, *a*, as the corresponding Jaccard distance:

$$p_{\mathrm{accessory}}=(1-a).$$

**Figure 1.**
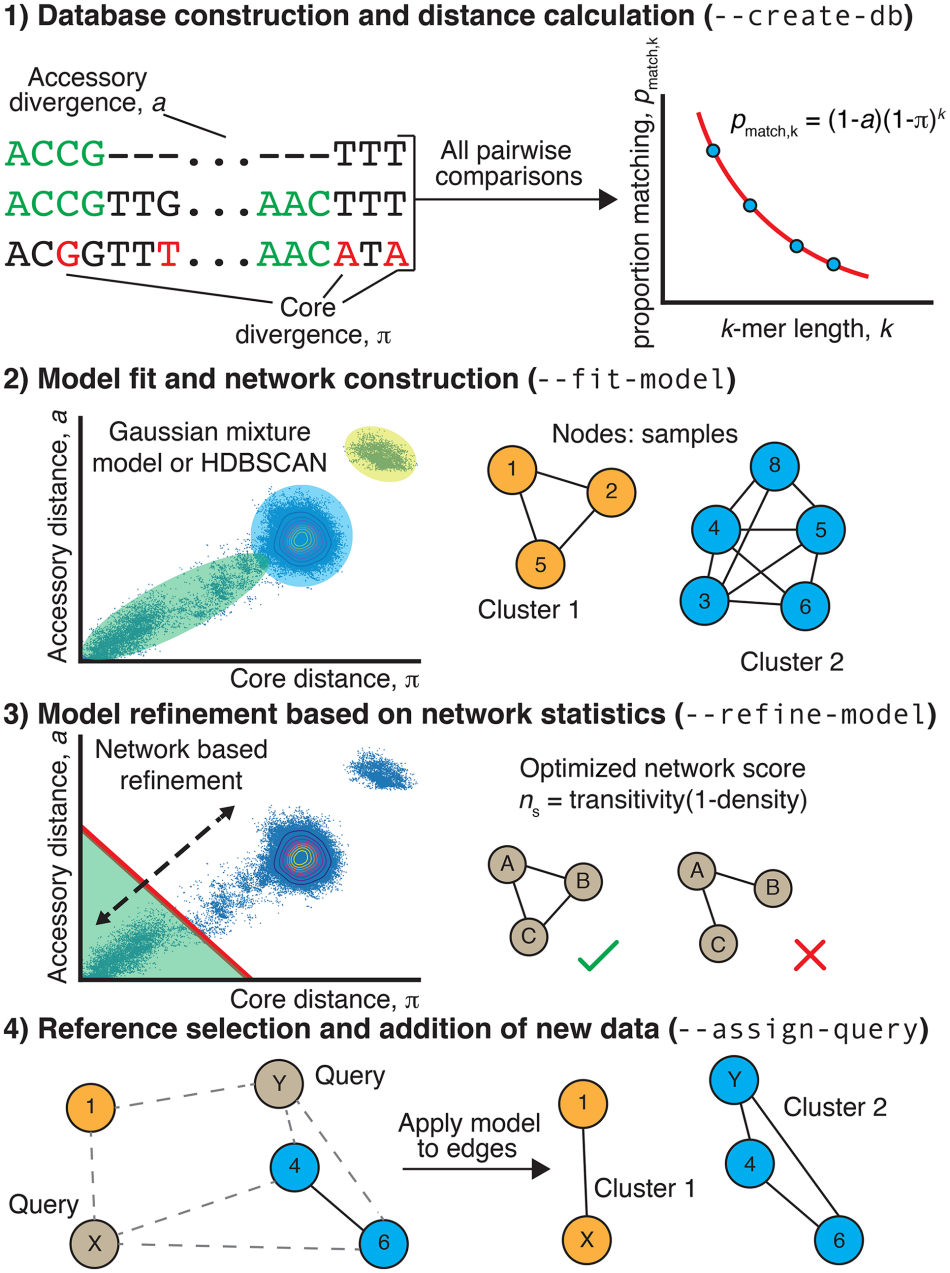
Summary of the PopPUNK algorithm. (Step 1) For each pairwise comparison of sequences, the proportion of shared *k*-mers of different lengths is used to calculate a core and accessory distance. Differences in gene content cause *k*-mers (examples highlighted in green) to mismatch irrespective of length, whereas point mutations distinguishing orthologous sequences cause longer *k*-mers to mismatch more frequently than shorter *k*-mers. (Step 2) The scatterplot of these core and accessory distances is clustered to identify the set of distances representing “within-strain” comparisons between closely related isolates. A network is then constructed from nodes, corresponding to isolates, linked by short genetic distances, corresponding to within-strain comparisons. Connected components of this network define clusters. (Step 3) The threshold defining within-strain links is then refined using a network score, *n*_s_, in order to generate a sparse but highly clustered network. (Step 4) Finally, the network is pruned by taking one sample from each clique. The distances between new query sequences and references are calculated, and within-strain distances used to add new edges. The clusters are then reevaluated as in Step 3, with the nomenclature being kept consistent with the original reference cluster names.

Unlike *p*_core_, *p*_accessory_ is independent of *k*, allowing both π and *a* to be jointly estimated from the relationship between *p*_match,*k*_ and *k* (Fig. 1):

$$p_{\mathrm{match},k}=(1-a){\left(1-\pi \right)}^{k}.$$

**Figure 2.**
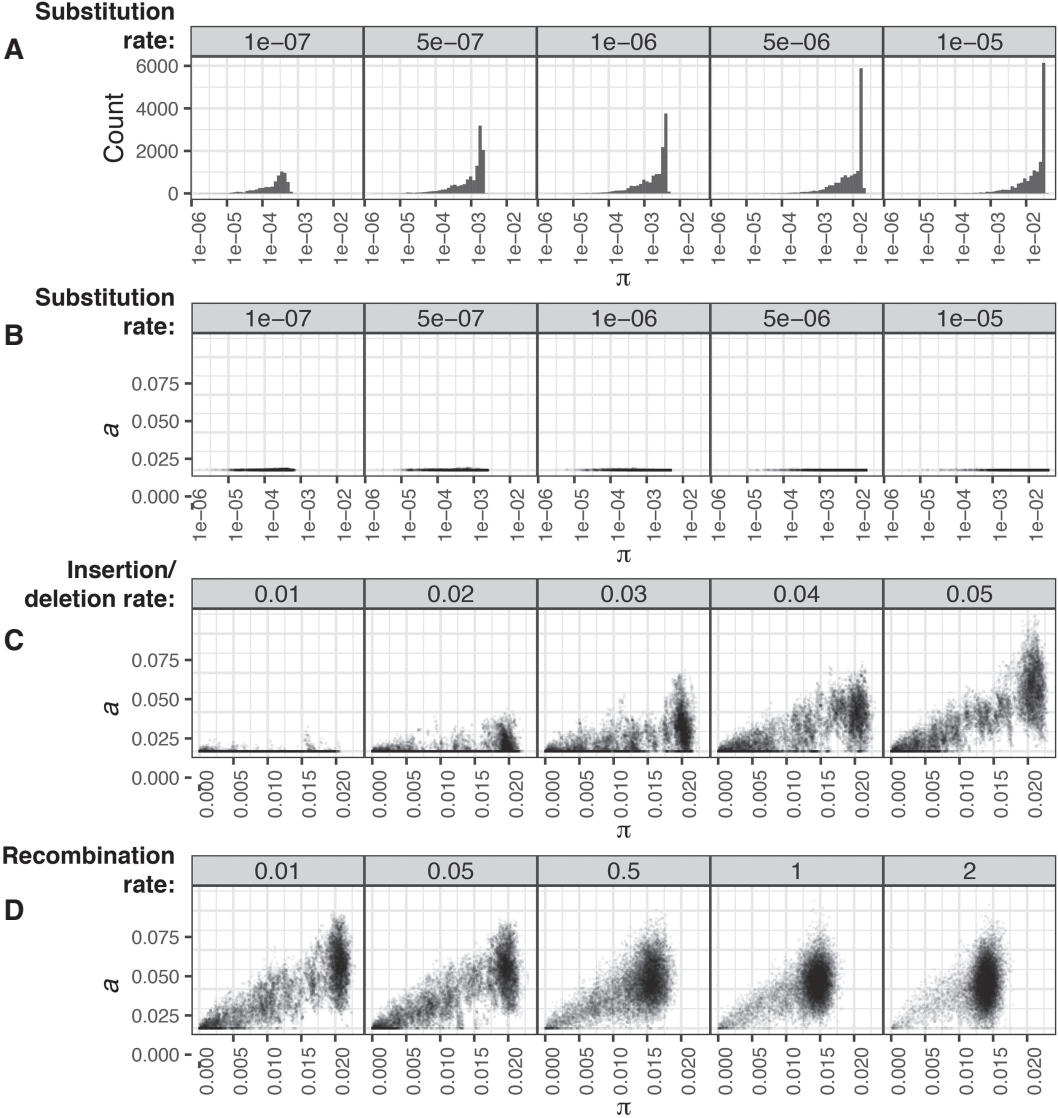
Detection of genetic diversity in simulated populations by PopPUNK. Each plot shows the deviation in gene sequence (π) and gene content (*a*) estimated by PopPUNK from a sample of 25 isolates from each of 50 simulations run with the same parameters. (*A*,*B*) Deviation through point mutation only. As the rate of point mutation (base^−1^ generation^−1^) was increased over two orders of magnitude, estimates of population-wide π increased accordingly, as shown by the distribution of pairwise core distances in the *top* row of histograms (*A*). The scatterplots *below* (i.e., in *B*) show that *a* measurements remained below 5 × 10^−3^, demonstrating the specificity with which divergence was measured. (*C*) Deviation through insertions and deletions. To test the estimation of *a* in a clonally evolving population, simulations included insertions and deletions of 100-bp segments occurring at a rate defined relative to the fixed point mutation rate of 5 × 10^−6^ base^−1^ generation^−1^. Estimates of *a* increased proportionately with this rate, without affecting the observed range of π. (*D*) Effects of recombination on the distribution of genetic diversity. With the insertion and deletion rate fixed at 0.05 relative to the point mutation rate of 5 × 10^−6^ base^−1^ generation^−1^, the rate of recombination relative to point mutations was then varied. This resulted in a concentration of the estimated distances into a single mode, representing the changing population structure as frequent exchange between isolates homogenizes the divergence between them in both gene sequence and content.

To test whether this approach was effective in differentiating core and accessory divergences, we performed forward-time simulations of bacterial populations diversifying through point mutations, large insertions and deletions (indels), and recombination using Bacmeta (Sipola et al. 2018) using the parameters listed in Supplemental Table S1. Those simulations in which sequences diverged only through point mutations all correctly identified *a* < 5 × 10^−3^, whereas π increased according to the set point mutation rate over multiple orders of magnitude, even in the presence of recombination (Fig. 2A,B; Supplemental Figs. S1–S3). Further tests found PopPUNK could resolve genomes distinguished by only a single point mutation, with the *k*-mer sketch size used determining the balance between precision and computational speed (Supplemental Text S1; Supplemental Fig. S4). To test the accuracy with which *a* could be estimated, the point mutation rate was fixed at 5 × 10^−6^ bp^−1^ generation^−1^, and indels of a fixed size of 100 bp occurred at varying rates relative to point mutations to emulate changes of gene content. The calculated *a* covaried with the indel rate, without substantially affecting the inferred distribution of π (Fig. 2C; Supplemental Figs. S1, S2). When the indel rate was fixed at 0.05, the distributions of both *a* and π converged toward a single mode as the rate of exchange through recombination was increased (Fig. 2D), consistent with changes in the analogous core and accessory genetic distances observed in a study using a different framework to study the effects of sequence exchange (Marttinen et al. 2015). Hence, PopPUNK's use of variable-length *k*-mers can resolve variation in the genome content and sequence to generate a pairwise distance distribution that accurately reflects the population-wide distribution of genetic diversity.

### PopPUNK identifies divergence between bacterial genomes across multiple species

**Figure 3.**
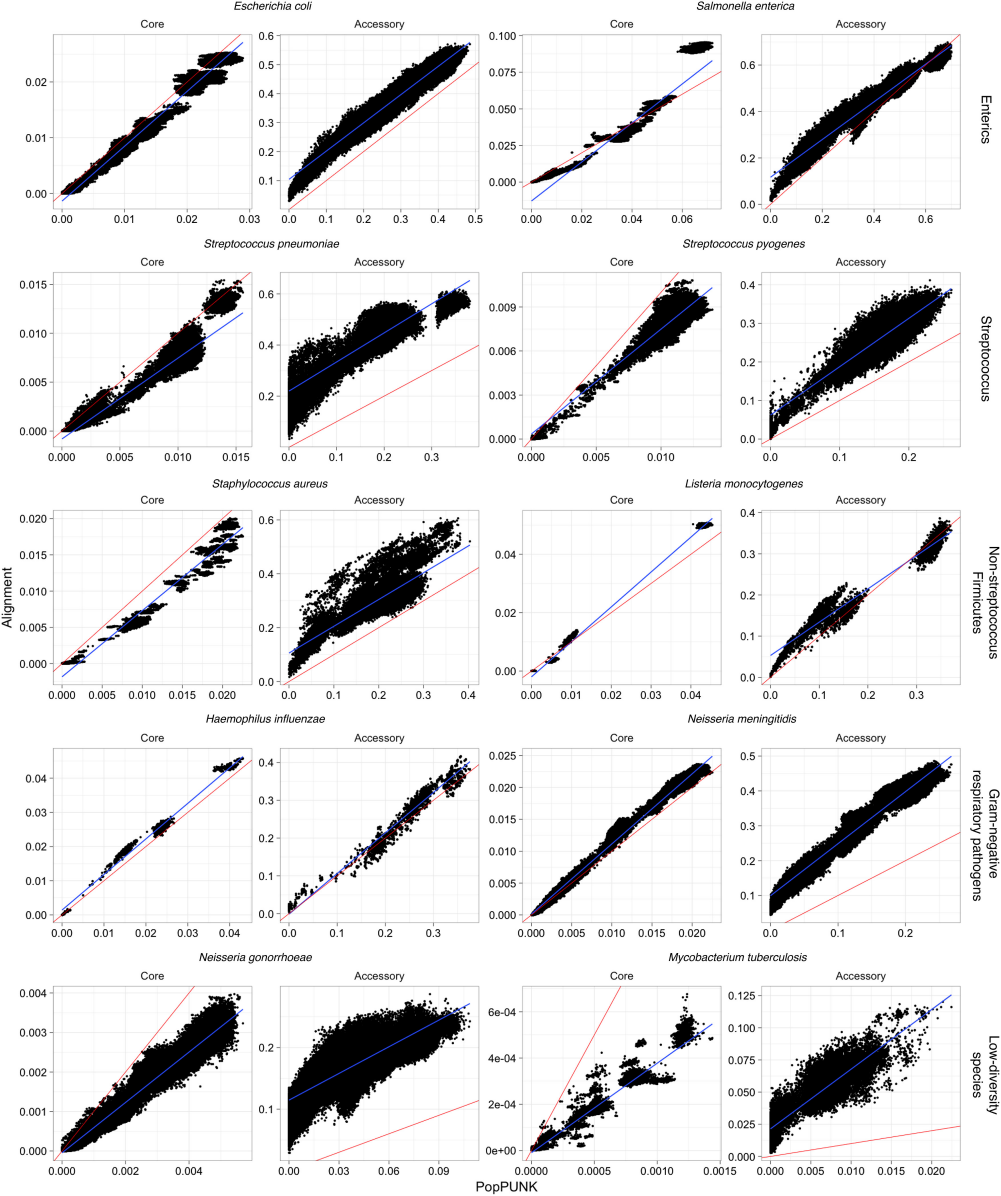
Comparison of core and accessory distances from PopPUNK (*x*-axis) and pan-genome construction with Roary (*y*-axis). For each species, the core distance was calculated as the Tamura-Nei (tn93) distance from the core genome alignment; the accessory distance was calculated as the Jaccard distance between binary strings representing gene presence/absence. In each panel, the line of identity (red line) and a linear regression (blue line) are also plotted. Sketch sizes were 10^4^, except for *M. tuberculosis* which used a sketch size of 10^5^.

To test whether PopPUNK could also produce accurate estimates of *a* and π when applied to real high-throughput sequencing data, the software was next applied to recent population genomics studies from 10 diverse bacterial species using the default sketch size of 10^4^. These were chosen to be genetically and ecologically varied, including enteric bacteria (*Escherichia coli* and *Salmonella enterica*), Gram negative respiratory pathogens (*Haemophilus influenzae* and *Neisseria meningitidis*), streptococci (*Streptococcus pneumoniae* and *Streptococcus pyogenes*), other Firmicutes pathogens (*Staphylococcus aureus* and *Listeria monocytogenes*), and two species in which limited genetic diversity has previously been detected (*Neisseria gonorrhoeae* and *Mycobacterium tuberculosis*). The pangenomes of these data sets were defined using Roary (Page et al. 2015), from which the population-wide core genome was aligned and pairwise distances calculated using the Tamura-Nei (tn93) distance (Tamura and Nei 1993). These pairwise distances use only loci conserved across at least 99% of the isolates in each sample, rather than the pairwise definition of the core intrinsic to PopPUNK. The genome content divergence was measured as pairwise Jaccard distances based on the presence and absence of coding sequences. In all cases, there was a strong linear correlation across the full range of both *a* and π (Fig. 3; Table 1). For π, the linear relationship was close to the identity line, indicating PopPUNK was accurately estimating the per-base probability of sequence divergence. The exception was *M. tuberculosis*, for which the range of π was an order of magnitude lower than in other species. To capture these differences PopPUNK needed to be run with a sketch size of 10^5^. Even with a 10-fold sketch size increase above the default, the analysis remained fast and memory efficient (Supplemental Table S2; Supplemental Fig. S5), and the estimated π values strongly correlated with the tn93 distances.

**Table 1.**
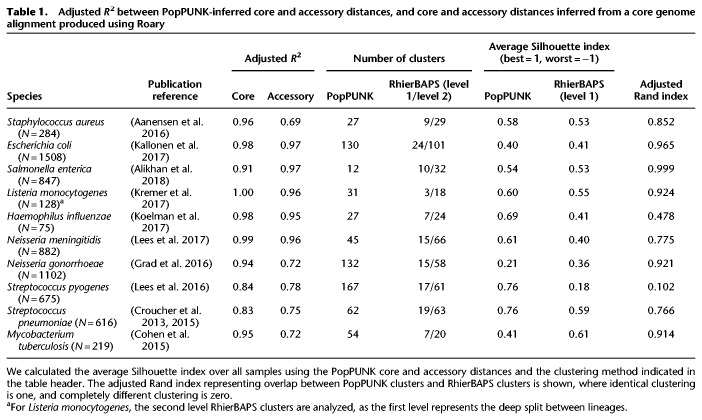
Adjusted *R*^2^ between PopPUNK-inferred core and accessory distances, and core and accessory distances inferred from a core genome alignment produced using Roary

The comparison of *a* with the pairwise gene content distances from Roary found close agreement in the cases of *H. influenzae*, *L. monocytogenes*, and *S. enterica*, but generally PopPUNK's estimates of accessory divergence were correlated with, but lower than, Roary's. Much of this is likely attributable to differences in the levels of within-species divergence between orthologs, which were not accounted for in the Roary analyses, which all used the default BLAST identity threshold (95%). However, PopPUNK enforces changes to the *k*_min_ value that determines how variation is split between π and *a* based on genome size. This dependence on the method used to identify orthologs could be demonstrated using the *S. pneumoniae* data set, in which there is a high divergence between the Roary and PopPUNK analyses. The smaller *a* estimates from PopPUNK were very similar to those estimated by an independent annotation and analysis of gene content (Supplemental Fig. S5) using COGtriangles (Kristensen et al. 2010; Croucher et al. 2014) rather than Roary. PopPUNK is still likely to be more conservative in calculating *a* than any ortholog-based analysis, as it will align any segments of genes that are similar, regardless of synteny, and the sketches it generates are nonredundant, meaning *a* is independent of repeat copy number.

The benefits of this are evident from the discrepancy between the distribution of *a* in *M*. *tuberculosis*, inferred to reach up to ∼10% by Roary but only ∼1.5% by PopPUNK (Fig. 3). *M. tuberculosis* exhibits little diversity in gene content, but ∼10% of the genome comprises repetitive, variable PE/PPE repeats (Cole et al. 1998). Additional analysis of complete, reannotated *M. tuberculosis* genomes also found this discrepancy in *a*, indicating the difference was not a consequence of inconsistent draft assemblies (Supplemental Fig. S6A,B). Instead, the discrepancy was approximately halved when Roary no longer used synteny to define orthologs (Supplemental Fig. S6C,D) and pairwise alignments with NUCmer (Delcher et al. 2003) identified similar proportions of divergent sequence as PopPUNK (Supplemental Fig. S6E). This was consistent with manual inspection of alignments, which suggested the accessory variation identified by Roary was largely a consequence of the difficulty of identifying some orthologs, rather than genuine differences in sequence content, largely as a consequence of expansion, contraction, and rearrangement of PE/PPE repeats. Nevertheless, PopPUNK can detect potentially biologically important divergence in intergenic regions (Oren et al. 2014), as it does not depend on genome annotation. Additionally, PopPUNK was between six- and 200-fold faster than Roary, while using between five- and 45-fold less memory (Supplemental Table S2). Therefore, PopPUNK is an efficient means of accurately measuring SNP and gene content divergence in species-wide genomic data sets.

### PopPUNK successfully resolves diverse bacterial populations into strains

**Figure 4.**
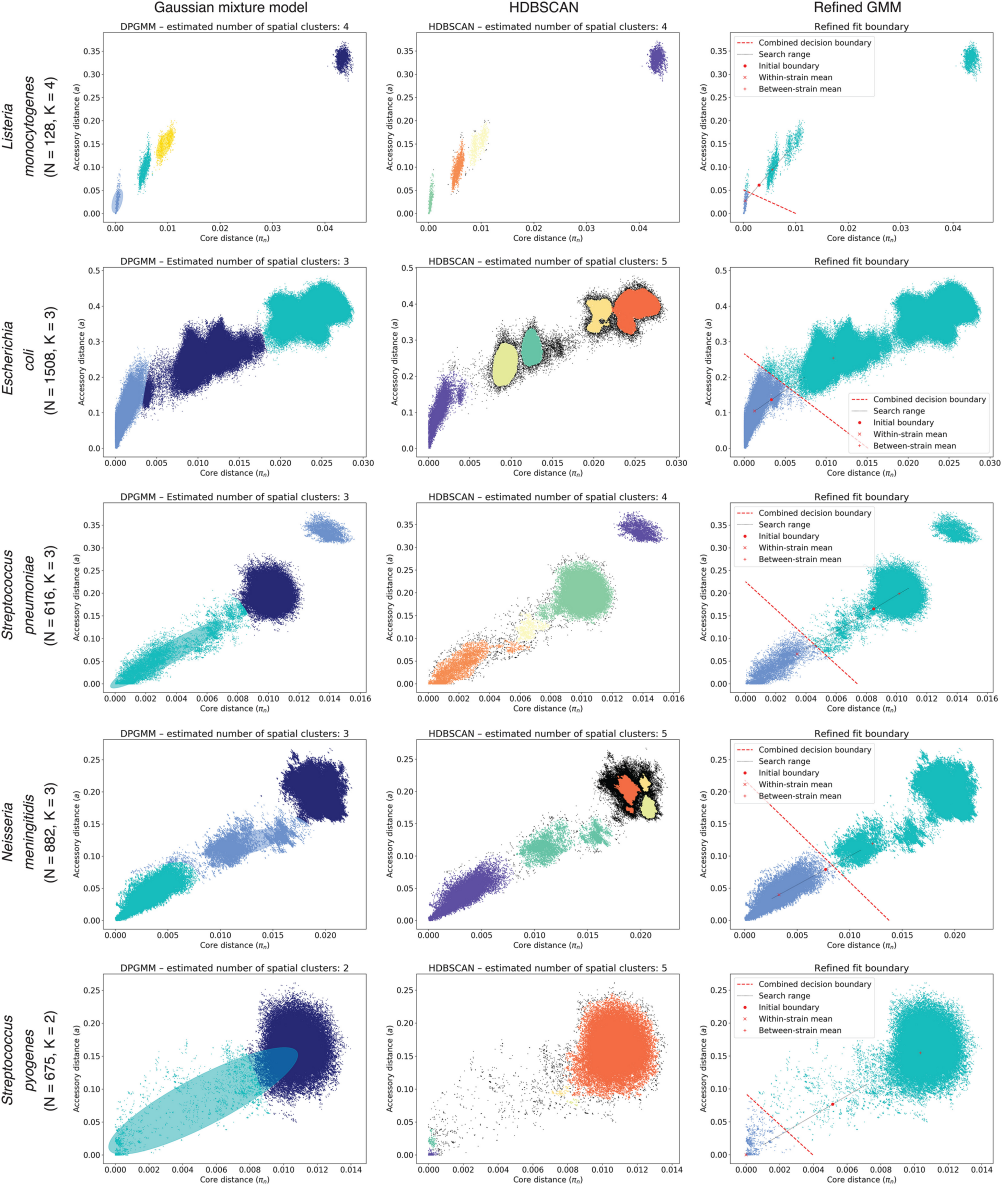
PopPUNK model fitting output for five archetypal examples (other species shown in Supplemental Fig. S8). Each row is a species, with each plot showing the distribution of core and accessory distances. In each plot, points are colored by their predicted cluster, and the cluster closest to the origin is the within-strain cluster. The two-dimensional Gaussian mixture model (2D GMM) is in the *left* column, which also shows ellipses with the mean and covariance of the fitted mixture components. The HDBSCAN plot in the *center* column shows unclassified noise points in black. The *right* column shows the fits when maximizing the network score to refine the 2D GMM fit. *Listeria monocytogenes* has clearly separated clusters, which were well-predicted by all methods. Although there is more complex structure on the plots, *Escherichia coli* and *Neisseria meningitidis* have a within-strain cluster also well captured by all approaches. In *Streptococcus pneumoniae*, recombination makes the boundary between clusters less distinct, and the mixture model includes too many links (Fig. 5A). HDBSCAN is more accurate, but the refinement of the initial fit provides the most accurate and intuitive demarcation of the within-strain links. *Streptococcus pyogenes* exhibits low within-strain recombination; hence, it has a dense cluster of points near the origin of the graph, but high between-strain divergence, resulting in the single, broad between-strain set of points. Network score fit refinement is required for an accurate model fit in this case.

PopPUNK successfully replicated the discontinuous distribution of *a* and π between all pairs of sequences in an *S. pneumoniae* population (Supplemental Fig. S7), which was previously shown to reflect the sequence clusters within the population (Croucher et al. 2014). Bacterial populations can be considered as being composed of multiple strains if there is a separation between the shorter genetic distances, corresponding to within-strain comparisons, and the larger between-strain distances, which may form one or more clusters in the plot (Fig. 2). To test whether this also applied to other bacterial pathogens, the pairwise *a* and π distributions were plotted for the other nine species-wide collections listed in Table 1 (Fig. 4; Supplemental Fig. S8). Species known to exchange sequence through homologous recombination at similar frequencies to *S. pneumoniae*, such as *Neisseria meningitidis* (Fig. 4), exhibited a similar distribution of pairwise genetic distances. The group of within-strain pairwise distances, found near the origin of the graph, was elongated, likely as a consequence of extensive diversification of strains through transfer of genomic islands and shuffling of core sequence through homologous recombination. The between-strain distances were primarily concentrated within a single dense modal cluster, consistent with the simulations involving high level of recombination in Figure 2. In *S. pneumoniae*, inclusion of *a* distances clearly identified a third component comprising comparisons with atypical unencapsulated isolates (Croucher et al. 2014).

In contrast, *Streptococcus pyogenes* strains exhibit little evidence of recent diversification through homologous recombination (Nasser et al. 2014), hence, the within-strain distances were tightly clustered near the origin of the graph (Supplemental Fig. S8). Nevertheless, between-strain distances remained concentrated in a single node, consistent with the much higher level of recombination inferred across broader samples (Didelot and Maiden 2010). A different pattern was evident in other species in which homologous recombination is infrequently observed, such as *Escherichia coli*, *Salmonella enterica*, and *Staphylococcus aureus* (Fig. 4; Supplemental Fig. S5). These exhibited much stronger evidence of deep population structure, characterized by multimodal between-strain distance distributions that likely reflect ancestral divergences that have not been overwritten by sequence exchange. The distribution of within-strain distances had high variance in the accessory direction, which is likely to reflect rapid diversification in gene content through movement of mobile genetic elements in the absence of core genome diversification through homologous recombination (Zhou et al. 2014; Aanensen et al. 2016; Kallonen et al. 2017). A more extreme version of this pattern was clear in *Listeria monocytogenes* and *Haemophilus influenzae*, which are composed of deep-branching lineages that result in small, tight clusters being formed in the π-*a* distance space. Although π and *a* were generally correlated, the inclusion of accessory distances helped further resolve clusters of pairwise comparisons into separate components, both permitting easy identification of atypical samples likely representing contaminated or low-quality assemblies, and providing a clear overview of sequence distribution in the species. Hence, PopPUNK can identify evidence of multistrain populations across a taxonomically diverse set of species with varied ecologies and rates of horizontal sequence exchange.

### PopPUNK can exploit network properties to refine strain definitions

**Figure 5.**
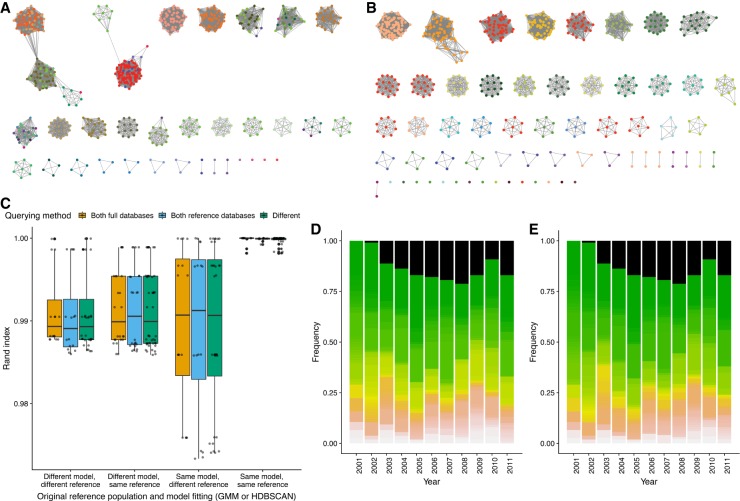
Network and query assignment for *S. pneumoniae* and *E. coli*. (*A*) Cytoscape view of the network for the Massachusetts *S. pneumoniae* data set using the 2D GMM fit. Nodes (colored dots) are samples and edges (lines) are those pairwise distances classified as within-strain. The nodes are colored by clusters according to the refined fit in *B*, showing which clusters are incorrectly merged in the mixture model fit. (*B*) As in *A*, but showing the network after fit refinement. High-stress edges causing clusters to be merged have been removed after maximizing the network score. (*C*) Box plots showing the similarities between cluster assignment when running PopPUNK in different modes. The different model types (2D GMM or HDBSCAN) implemented in PopPUNK were each fitted to either the Massachusetts or Maela *S. pneumoniae* population defined in Corander et al. (2017), then refined. The three nonreference populations were then added in successive batches, either through comparisons to the full data set or a representative set of reference sequences selected based on network structure, in all possible permutations. The Rand index was used to quantify clustering similarity between all those permutations in which the final population to be integrated was the same; only those isolates in the most recent extension of the network were used. These values are shown separated according to the starting reference population (Massachusetts or Maela), initial model (2D GMM or HDBSCAN), and comparison method (bar color; full database or references only). (*D*,*E*) Simulating surveillance of the *E. coli* BSAC population. A five-component 2D GMM was fitted to the pairwise distances between the 2001 isolates, and batches of isolates from successive years added sequentially either retaining the full database throughout (*D*) or identifying references after each addition (*E*). The stacked bar charts show the prevalence of strains in the population in each year, with the black component representing isolates of the multidrug-resistance-associated ST131 lineage, which emerged from 2002 onward. The full output of this analysis is provided in Supplemental Table S3.

In order to identify clusters in π-*a* space, two alternative approaches were implemented within PopPUNK: two-dimensional Gaussian mixture models (2D GMMs), which split the points into a user-specified maximum of *K* two-dimensional Gaussian distributions; or HDBSCAN, which is run iteratively to identify fewer than a user-specified maximum number of clusters *D* (Methods). The results of the application of both methods to the genomic data sets listed in Table 1 are shown in Figure 4 and Supplemental Figure S8: In each case, both methods were successfully able to resolve the pairwise distances into discrete clusters, of which the closest to the origin represent within-strain relationships. The bacterial population can then be represented as a network in which each node corresponds to an isolate, and each within-strain relationship to an edge between these nodes (Fig. 1). This network has the property that strains can be defined as the separate connected components (Fig. 5).

Neither the 2D GMM or HDBSCAN methods alone could satisfactorily resolve the recombinogenic populations into strains, primarily due to the diffuse nature of the within-strain distribution, which likely reflects the heterogenous rates of diversification observed in different strains (Croucher et al. 2013; Didelot et al. 2013). For the 2D GMMs, this was manifested as insufficiently specific, elongated within-strain distributions, which incorrectly included between-strain links as edges. For HDBSCAN, the expectation of a background noise in the distribution meant some within-strain points were omitted from the appropriate cluster. Only a few spurious connections can have a dramatic effect on strain definitions, as previously observed for MLST clonal complexes (Turner et al. 2007). For strain definitions to be robust, networks should have a nonoverlapping community structure, with distinct components that are highly internally connected. To achieve this, a linear threshold in π-*a* space was used to define the genetic divergence below which edges would link isolates (Fig. 1). Varying this identified a transition point at which there was a rapid increase in the transitivity of edges in the network, and a corresponding decrease in edge density (Fig. 5A), as spurious high-stress edges linking highly connected components are eliminated (Supplemental Figs. S9, S10). Therefore, a network score statistic *n*_s_ ranging between zero and one was defined:

$$n_{s}=\mathrm{transitivity}\times (1-\mathrm{density}).$$

For each data set, *n*_*s*_ was first calculated by using a linear boundary to separate the within and between-strain distances, based on the 2D GMM of HDBSCAN results. This boundary's position was then optimized to maximize *n*_*s*_ (Methods). This provided an intuitive threshold defining within-strain distances (Fig. 4) and tended to be consistent whether initialized from either a 2D GMM or HDBSCAN (Supplemental Fig. S7). Inspection of the network before and after this refinement showed that small numbers of spurious edges between high frequency clusters were removed, and low-frequency clusters were kept distinct (Fig. 5B), increasing the robustness of strain definitions.

### Comparison of PopPUNK with alternative typing methods

For the populations listed in Table 1 the strain definitions resulting from model refinement were evaluated relative to the top level clusters identified from the core genome alignment using RhierBAPS (Tonkin-Hill et al. 2018). PopPUNK used 15- to 74-fold less memory and ran between 10- and 100-fold faster than RhierBAPS (Supplemental Table S2). The biggest differences in performance were observed in relatively small collections containing extensive core genome divergence (Fig. 3), presumably representing the complexity of fitting the RhierBAPS model to such data. The number of clusters estimated by each method was similar (Table 1). The adjusted Rand index can be used to compare the clustering results. This ranges from zero (different clusters) to one (the same clusters) while adjusting for chance cluster overlap (Rand 1971; Hubert and Arabie 1985); the average adjusted Rand index of 0.852 indicated a high level of overlap between the methods. Based on the Silhouette distance calculated from the π and *a* distances, the clustering identified by PopPUNK was typically of similar, or better, quality than that of RhierBAPS. For instance, in the case of *S. enterica*, the clusters identified agreed perfectly with a recent reappraisal of species and subspecies definitions (Alikhan et al. 2018), whereas using RhierBAPS leads to two cases of subspecies being merged, and a cluster with a single member being added to another larger cluster.

Notably, RhierBAPS produced a superior clustering for *N. gonorrhoeae* and *M. tuberculosis*, which lack the assumed strain structure (Supplemental Fig. S8). For the analyses of such species, or individual strains within multistrain species, PopPUNK allows for models to be refined separately for the π and *a* distances (Supplemental Text S1). Using this approach to analyze both the *S. pneumoniae* multidrug-resistant lineage PMEN14 and *N. gonorrhoeae* found these core and accessory clusterings to be highly discrepant. For PMEN14, this was consistent with the frequent infection by phage previously identified using PANINI (Abudahab et al. 2018), whereas for *N. gonorrhoeae*, pairs separated by high *a* but low π were found to be distinguished by the Gonococcal Genomic Island through association analysis using pyseer (Supplemental Fig. S11; Lees et al. 2018a).

To further evaluate the clusters generated by PopPUNK using the models refined jointly on π and *a*, they were compared to SNP distances calculated from the Roary core genome alignments (Supplemental Table S4; Supplemental Fig. S12) and the corresponding maximum likelihood phylogenies (Supplemental Table S5). In general, both the PopPUNK and RhierBAPS clusterings corresponded to clades in the phylogenies, indicating PopPUNK strains are typically related by common descent. Calculating the within-strain SNP distances, which approximates the SNP distance cutoff that could be used to define sequence clusters, varied by more than two orders of magnitude between species. This demonstrates the flexibility of PopPUNK's approach, which can adapt to the varied distance distributions across species, rather than favoring a particular threshold.

The PopPUNK clusters were also compared to MLST and cgMLST schemes for two taxonomically diverse species with good quality typing schemes, *L. monocytogenes* and *E. coli* (Supplemental Tables S6, S7). The methods used, stringMLST (Gupta et al. 2017) and chewBBACA (Silva et al. 2018), were both more resource intensive than the PopPUNK analyses. For direct comparison with the PopPUNK strains, clonal complexes were defined using different threshold numbers of allele differences. In both species, the PopPUNK clusters were highly similar (adjusted Rand index above 0.98) to MLST clonal complexes defined by linking only single locus variants. For the cgMLST schemes, the PopPUNK clusters were highly similar to clustering samples with up to 100 allele differences in the 1701 core genes of *L. monocytogenes* (adjusted Rand index = 0.974) and up to 1000 allele differences in the 2360 core genes of *E. coli* (adjusted Rand index = 0.997). Hence, PopPUNK efficiently identifies similar relationships to cgMLST approaches, but can automatically adapt its clustering threshold according to the studied population, generating better average Silhouette distances in π-a space than gene-by-gene methods.

### PopPUNK rapidly integrates new genomic data into clusters

By first generating a reference database and defining a model by which *a* and π distances can be assigned as being within- or between-strain, PopPUNK allows the network by which strains are defined to be extended. New batches of genomes can then be included without needing to refit the model or recalculate all pairwise distances. This can result in existing strains expanding in number, merging with others, or new strains not previously represented in the database being founded. By automatically rebuilding an updated database, PopPUNK allows iterative expansion through addition of successive batches of genomes. The accuracy of this approach was tested using a data set of 4107 draft *S. pneumoniae* genomes resulting from the combination of four different populations, known to have divergent strain compositions (Supplemental Fig. S13), sampled from Massachusetts (USA), Southampton (UK), Nijmegen (Netherlands), and Maela (Thailand) (Corander et al. 2017). Both the 2D GMM and HDBSCAN models were fitted and subsequently refined based on network properties using either the Massachusetts or Maela collections as the initial reference set. The three nonreference populations were then added as individual batches in every possible permutation to test the consistency of clusters from different starting points. The final clusterings of the isolates in the last population to be added were compared using the Rand index (Fig. 5C) and the adjusted Rand index (Supplemental Fig. S14). Using the same reference population and refined model, when the strains of the final population were compared with the middle two populations having been included in different orders, the Rand indices were all above 0.9997 (Fig. 5C). Comparisons were also made between successive queries assigned using different initial reference populations (Massachusetts or Maela) or model fits (GMM or HDBSCAN). The consistency of the π-*a* distributions, and refined model fits, meant there was only a slight decrease in the reproducibility of the clustering, with the median Rand index still greater than 0.99 (Fig. 5C). These were all highly similar to the results obtained when all 4107 isolates were clustered in a single step, regardless of which model fitting approach was used (Supplemental Fig. S15). The clustering observed after successive additions of query sequences to the network of genomes therefore exhibits reassuringly little sensitivity to the original choice of population and model fit.

The addition of batches by calculating the distance to every sample in the original clustering is inefficient, as the tight clusters of isolates within the same strain will each be separated from a given query by similar π and *a* distances. By default, PopPUNK reduces a full database to a set of reference genomes, which includes just one representative from each clique (i.e., a fully connected component) within the network (Fig. 1). This selects at least one isolate from each strain cluster to use as a reference and will include multiple representatives of clusters that are not fully transitive, such that any new within-strain query will have at least one edge connecting it to the correct cluster. This allows for faster and more efficient analysis of new batches of data, from which new references can be extracted. To test whether this approach caused any decrease in clustering reproducibility, it was used to successively integrate the four test populations of *S. pneumoniae* as before (Fig. 5C). No decline in the accuracy of isolate assignment to strains was detected compared to using the full databases, nor was there a decrease in similarity to the global clustering of all populations (Supplemental Fig. S15). The final networks of all four combined populations contained a median of 281.5 references (range: 111–477): This almost 15-fold reduction in the database size resulted in a median 4.6-fold decrease in CPU time required for the addition of the final batch.

To test how this worked in a surveillance setting, the BSAC *E. coli* collected between 2001 and 2011 (Kallonen et al. 2017) were analyzed in batches, according to their year of isolation. A refined GMM model was fitted to the 2001 data set, and later years added either using the full database, or a reference set updated after each batch addition. This approach found similar population trends to the published analysis (Fig. 5D). The multidrug-resistance-associated ST131 lineage identified as a new strain by PopPUNK as soon as it was first detected in 2002, with all later representatives assigned to this cluster. Results using the full and reference-only approaches were always similar, and often identical, despite the latter approach reducing CPU and memory use by ∼50% (Fig. 5E). There was no trend toward greater divergence over time, as the addition of data allowed some closely related strains to be merged (Supplemental Table S3). Hence, using this network-based design, PopPUNK can efficiently expand an accurate database as new data become available.

### PopPUNK outputs can be used directly for interactive browser-based analysis

PopPUNK can combine input epidemiological data with the genetic analysis results to generate files for visualization and analysis with the online visualization software Phandango (Hadfield et al. 2018), the browser-based viewer GrapeTree (Zhou et al. 2018), network analysis tool Cytoscape (Shannon et al. 2003), and the online epidemiology platform Microreact (Argimón et al. 2016). Microreact allows interactive views of a neighbor joining tree of core distances on the left and a t-SNE projection of accessory distances on the right, using Microreact's network interface developed for PANINI (Abudahab et al. 2018), with nodes in both colored by PopPUNK strain assignment. Links and descriptions of examples of such analyses are provided in Supplemental Table S8, including examples of how PopPUNK can highlight newly added data relative to the existing strains. As these platforms allow visualization at a resolution finer than that of overall clusters, within-strain structure can be discerned, such as the three clades of the *E. coli* ST131 lineage (https://microreact.org/project/rJGAHaPtm) (Petty et al. 2014), and the accessory variation in *S. pneumoniae* PMEN14 (Supplemental Text S1; Supplemental Fig. S16; Abudahab et al. 2018). This also allows outbreak data to be analyzed, visualized, and shared online in a few minutes (Supplemental Text S1). PopPUNK thereby provides a simple and efficient means to intuitively and interactively analyze complex data using a platform that facilitates online collaboration and publication.

## Discussion

The complexity and scale of bacterial genomic epidemiology data sets necessitates new approaches for population-wide analyses. PopPUNK responds to these needs by providing a comprehensive suite of algorithms for analyzing large bacterial population genomic data sets, from species-wide collections to outbreak studies, overcoming the technical and computational limitations of previous approaches. The method's speed results from the memory- and CPU-efficient estimation of core and accessory pairwise distances between isolates using MinHash-optimized *k*-mer comparisons. The strains identified by PopPUNK are highly consistent with the clusterings generated by RhierBAPS, MLST, and cgMLST, as well as the clades in maximum likelihood core genome phylogenies. The annotation- and alignment-free approach means running PopPUNK on draft assemblies is up to 200-fold faster than Roary, which starts from annotations; up to 100-fold faster than RhierBAPS, which starts from a core genome alignment; and up to threefold faster than cgMLST, which starts from a predefined scheme. The use of *k*-mer comparisons also fully exploits the information available in the entire genome assembly, without being limited to the core or a predefined set of common loci, allowing variation in the accessory genome to be quantified simultaneously. Unlike gene-by-gene methods, PopPUNK can be run on a new species or collection without necessitating careful definition and evaluation of a typing scheme. The software can also adapt to using different genome sizes, through altering *k*_min_, and different levels of sample-wide diversity, by altering the MinHash sketch sizes. Additionally, there is flexibility in its application of machine learning techniques for defining strains, making it applicable across a wide variety of population structures and collection sizes, and providing the opportunity to expand the implemented repertoire of these techniques used by the software.

Model refinement can also be adapted to the observed π-*a* distributions. The concordance of PopPUNK's default strain definition with clusters identified by other methods is consistent with them representing coherent natural populations (Alikhan et al. 2018), validating their critical use epidemiologically both for following longitudinal trends and understanding the distribution of clinically important traits in cross-sectional samples. The clusters are typically comparable to single MLST allele differences, although with the advantage that the boundary to merge clusters is automatically found through network refinement, and core and accessory distances are immediately available for more detailed within-strain investigation. Where multistrain structures are not evident in a sample, PopPUNK can divide collections into sequence clusters, using the divergence in the core genome (Palys et al. 1997), or genomotypes, defined as being similar in the accessory loci they harbor (Doolittle 2002). In each mode, PopPUNK applies a stringent threshold that minimizes the probability of spurious links, which connect nodes with high stress, and eventually lead to “straggly” clusters indirectly linking distantly related isolates. This approach also avoids the problem of clusters arising from diverse sets of rare genotypes, rather than lineages descended from a recent common ancestor (Croucher et al. 2013; Grad et al. 2016; Willemse et al. 2016). PopPUNK instead separates these into multiple small, distinct groupings, allowing emerging genotypes to be identified rapidly, emulating one of the most desirable properties of a high-quality MLST scheme.

The stringency of the within-strain threshold also ensures strain definitions are persistent, and therefore robust to the addition of further batches of data, as demonstrated for both *S. pneumoniae* and *E. coli*. PopPUNK is therefore ideally suited to addressing the current limitations of *k*-mer-based epidemiological methods, which suffer from the absence of an appropriate curated database or a stable strain nomenclature (Nadon et al. 2017), while retaining the benefit of being scalable to tens of thousands of genomes. Furthermore, by exploiting network properties to identify a reduced set of informative references, PopPUNK is much faster than naive *k*-mer comparison approaches when data are added in batches, as in epidemiological surveillance applications. Such functionality is further enhanced through outputs that can be readily shared using online visualization and analysis tools. Hence PopPUNK can be used as a pipeline to rapidly perform the most important tasks in bacterial epidemiology: analyzing population structure, identifying strains, finding substructure within these clusters, and integrating new data. Alternatively, its modularity means individual components may be used standalone, for example, when opting to use traditional read mapping to perform detailed analysis of variation with a cluster, or using allelic instead of core distances to construct clusters through the network. PopPUNK therefore greatly expands our capacity to conduct genomic surveillance of bacterial pathogens.

## Methods

### Rapid calculation of core and accessory divergences

PopPUNK uses pairwise comparisons through *k*-mer matching between two sequences (*s*_1_ and *s*_2_) at multiple *k* lengths to distinguish divergence in shared sequences, π (Nei and Li 1979), from divergence in accessory locus content, here defined as *a*, the Jaccard distance between the sequence content:

$$a(s_{1},s_{2})=1-\frac{\left(s_{1}\;\cap \;s_{2}\right)}{\left(s_{1}\;\cup \;s_{2}\right)}.$$

For any given *k*, a MinHash algorithm can efficiently estimate *a*, albeit with confounding by divergence due to π that prevents matching between *k*-mers in the core genome common to (*s*_1_,*s*_2_). Assuming that such sequence mismatches are distributed evenly throughout the genome, *a* can be estimated independently of *k* and π by calculating a function for each (*s*_1_,*s*_2_) pair that relates the proportion of shared *k*-mers *p*_match_, π, and *a* over a series of *k*-mer lengths *k*:

$$p_{\mathrm{match},k}=(1-a){\left(1-\pi \right)}^{k},$$

which we fit as a linear relationship in log space by minimizing the least squares divergence, and constraining *a* > 0; π > 0:

$$\log(\;p_{\mathrm{match},k})=\mathrm{lo}g(1-a)+k\;\log(1-\pi ).$$

As both distance estimates are symmetrical, only a single comparison is calculated between each (*s*_1_,*s*_2_) pair, corresponding to the upper triangle of a square distance matrix, or (*n*-1) × *n*/2 comparisons. The calculation of all pairwise *p*_match_ are further optimized for speed in our implementation (Supplemental Methods). Supplying each input sequence as a FASTA-formatted assembly, we use Mash (Ondov et al. 2016) with a default sketch size of 10^4^ to efficiently calculate *p*_match_ for every second *k*-mer size from *k* = *k*_min_ to *k* = *k*_max_ (29, by default). We constrain *k*_min_ such that the probability of a random *k*-mer match *p*_random_ is less than 5% (Supplemental Methods):

$$k_{\min}=\frac{\log(L)+\log(1-p_{\mathrm{random}})-\log(p_{\mathrm{random}})}{\log(4)},$$

where *L* is the genome length and log(4) enters due to the alphabet size (assuming minimal gaps or unspecified bases). For typical bacterial genomes with *L* between 1 and 8 Mb, this corresponds to a *k*_min_ of either 12 or 13.

### Creating clusters using core and accessory divergences

To create clusters of strains from the distance pairs (π, *a*), we first perform spatial clustering on all pairwise distances within the sample set to attempt to find the within-strain component nearest the origin. This is achieved using either a Gaussian mixture model (GMM) with a set maximum number of components *K*, or HDBSCAN with a set maximum number of clusters *D*.

The distances defined as being within-strain are those in the component or cluster nearest the origin. We then create an undirected, unweighted network consisting of samples as nodes, linked by edges when the distance was classified as within-strain. We define PopPUNK clusters as the connected components of this network (Figs. 1, 5). New query samples are added by calculating the distances to samples in the reference network and adding in those edges predicted to be within-strain. Clusters are updated to reflect the connected components.

This network is also used to select representative references, comprising one or more samples from each cluster, by retaining only one sample from each clique in the network (where every node in a clique is mutually connected to every other node), thereby reducing its overall size with negligible loss of information (Fig. 5C; Supplemental Fig. S15). Finally, the GMM or HDBSCAN model fit can be improved by moving the within-strain boundary and using the resulting network's properties to calculate and maximize the network score *n*_s_. Full details of the PopPUNK clustering method and its implementation can be found in the Supplemental Materials.

### Output and visualization

Although a neighbor joining tree constructed using pairwise Jaccard distances directly has been shown to be reasonably accurate, using core genome divergence gives a more accurate tree topology (Lees et al. 2018b). We therefore use dendropy or RapidNJ to produce a midpoint-rooted neighbor joining tree from the core distances (Sukumaran and Holder 2010; Simonsen et al. 2011), and an implementation of t-SNE in the sklearn package (Pedregosa et al. 2011) to generate a projection of isolates based on the pairwise *a* matrix (Abudahab et al. 2018). To enable interactive visualization of these outputs, PopPUNK can write files formatted for Microreact (Argimón et al. 2016), Phandango (Hadfield et al. 2018), and GrapeTree (Zhou et al. 2018). Each of these can be automatically joined with other user-provided metadata for visualization. We also produce output for Cytoscape (Shannon et al. 2003) for inspection and analysis of the network.

### Comparison with other methods using both simulated and real data

To determine the specificity of PopPUNK in distinguishing core sequence divergence from differences in gene content, forward-time simulations were run using Bacmeta (Sipola et al. 2018). A population of 1000 bacteria, each represented by 100 loci each 1 kb long, was simulated for 1000 generations. Insertions and deletions were fixed at a length of 100 bp. Recombinations always exchanged a complete locus and were independent of sequence divergence between donor and recipient. A sample of 25 genomes was output from the final generation of each simulation, which were analyzed using PopPUNK using default settings. Pairwise distance estimates from 50 independent simulations were then combined for plotting.

To compare PopPUNK with other clustering methods, we selected a range of previously published data sets on 10 different bacterial species (Croucher et al. 2013, 2015; Cohen et al. 2015; Aanensen et al. 2016; Grad et al. 2016; Lees et al. 2016, 2017; Kallonen et al. 2017; Koelman et al. 2017; Kremer et al. 2017; Alikhan et al. 2018). For each data set, as well as PopPUNK, we ran Roary (Page et al. 2015) to construct a pan-genome, using a BLAST sequence identity cutoff of 95%. We calculated core distances using the Tamura-Nei (tn93) distances (Tamura and Nei 1993) in the core genome alignment. For accessory distance, we used the Jaccard distance between the accessory gene presence/absence vectors. For comparison with another high-performance clustering algorithm, we ran RhierBAPS using between 8 and 16 cores depending on data set size (Tonkin-Hill et al. 2018). We estimated the maximum cluster size by data set, using the output from Roary and information from published analyses of these data sets.

For each species, we generated a maximum-likelihood tree from the Roary core genome alignment SNPs using IQ-TREE v1.6.3 with a GTR + I + G + ASC model (Nguyen et al. 2015). For each of these trees, we also counted polyphyly for each nonsingleton cluster. We identified all pairs of isolates from the same cluster that shared a most recent common ancestor with any isolate from a different cluster. To quantify diversity within and between clusters, we calculated the SNP distance matrices from each core alignment using pairsnp (https://github.com/gtonkinhill/pairsnp). We selected and pruned the entries in the upper triangle, which correspond to within-cluster sample comparisons, leaving the remaining entries in the upper triangle corresponding to the between-cluster distances. For *E. coli* and *L. monocytogenes*, we also performed MLST and cgMLST assignment using stringMLST (Gupta et al. 2017) and chewBBACA (Silva et al. 2018), respectively. We used the database from EnteroBase for *E. coli* (Alikhan et al. 2018), and from Ridom for *L. monocytogenes* (Ruppitsch et al. 2015). We calculated the square symmetric matrix of pairwise allelic distances for each species and each scheme which, after applying an integer allelic distance cutoff, was then used as the input to PopPUNK to produce a network in the same way as with a π-*a* cutoff from network refinement.

### Software availability

Code is available on GitHub (https://github.com/johnlees/PopPUNK; Apache 2.0 license), through the Python package index (https://pypi.org/project/poppunk/), on Bioconda (https://anaconda.org/bioconda/poppunk) and as a tarball (Supplemental Data S1). Documentation can be found on readthedocs (http://poppunk.readthedocs.io/). Code used to perform additional analysis are also available on GitHub (https://github.com/johnlees/PopPUNK-scripts) and as a tarball (Supplemental Data S1). Online and interactive Microreact instances produced for each data set are listed in Supplemental Table S8. PopPUNK databases with the best model fits for each species can be downloaded from https://doi.org/10.6084/m9.figshare.6683624.

## Supplementary Material

Supplemental Material
