# Supplementary material for: Fast and flexible bacterial genomic epidemiology with PopPUNK
Source: Genome Res. 2019 Feb;29(2):304–16. doi: 10.1101/gr.241455.118 (PMC6360808; doi:10.1101/gr.241455.118)
Supplement: Supplemental Material [file supp_29_2_304__index.html]

Fast and flexible bacterial genomic epidemiology with PopPUNK — Fast and flexible bacterial genomic epidemiology with PopPUNK — Supplemental Material 

# Fast and flexible bacterial genomic epidemiology with PopPUNK

## Supplemental Material

- Supplemental\_Material.pdf
- Supplemental\_Data\_S1.zip
