# Supplementary material for: Fast and flexible bacterial genomic epidemiology with PopPUNK
Source: Genome Res. 2019 Feb;29(2):304–16. doi: 10.1101/gr.241455.118 (PMC6360808; doi:10.1101/gr.241455.118)
Supplement: Supplemental Material [file supp_gr.241455.118_Supplemental_Material.pdf]

Supplemental materials for

Fast and flexible bacterial genomic epidemiology with PopPUNK

John A. Lees, Simon R. Harris, Gerry Tonkin-Hill, Rebecca A. Gladstone,
Stephanie W. Lo, Jeffrey N. Weiser, Jukka Corander, Stephen D. Bentley,
Nicholas J. Croucher
Correspondence to: john.lees@nyumc.org and n.croucher@imperial.ac.uk

Contents

1 Text S1: Analysis of low diversity populations **2**

2 Supplemental methods **4**

3 Supplemental tables **7**

4 Supplemental figures **15**

Supplemental references **31**

1 Text S1: Analysis of low diversity populations

PopPUNK can operate at single nucleotide resolution

To determine the limits of resolution possible using PopPUNK, and therefore whether it could be used for surveillance of monomorphic pathogens or clonally-related outbreaks, distances were calculated between eight artificially-generated variants of a 2.2 Mb *Streptococcus pneumoniae* genome distinguished by three biallelic SNPs (Fig S3). This necessitates the use of a sufficiently large sketch size (10^5), which determines the coverage of the genome in the k-mer representation. With this increase even sequences distinguished by only a single SNP could be successfully resolved. This necessitates slightly longer runtimes, so we allow the user to select a larger sketch size than the default if there is a low level of divergence expected between isolates. Therefore given sufficiently high-quality data, PopPUNK can accurately resolve bacterial sequences at multiple scales of genetic divergence.

Using PopPUNK for outbreak analysis

The combined results arising from querying one dataset against another using PopPUNK can also be displayed using Microreact, GrapeTree, Phandango or Cytoscape. The additional isolates are highlighted in the output in each case, using 'Status' as a characteristic. This can be used for iteratively merging similarly-sized datasets, as illustrated in Fig 5C. As an example of such a merging in *S. pneumoniae*, the result of adding the Maela collection of ~3000 genomes into the Massachusetts collection of ~600 genomes is shown here: `https://microreact.org/project/SkZ23iPbX` (colours are clusters by default, colour by 'Status' to see whether the genomes are from the original reference population, or the added query population). This means PopPUNK can be applied as a rapid tool for ruling out potential outbreaks. As an example, we queried 175 *S. pneumoniae* isolates from the multidrug-resistant PMEN14 lineage (Croucher, Chewapreecha, et al., 2014) against the diverse Massachusetts species-wide carriage population sample of 616 isolates (`https://microreact.org/project/BkNqKdPb7`). This identified all the query isolates as belonging to a single strain, and generated a phylogeny in which they were confined to one clade and an accessory projection representing gene content differences, in less than six minutes using sixteen CPUs and less than 200 MB RAM. Repeating this analysis using an optimised reference database of 63 representative sequences and the same number of CPUs, the same process completed in under four minutes (`https://microreact.org/project/Hk-_FOoWX`).

PopPUNK can be used for analysis of low diversity pathogens

In the cases of *Neisseria gonorrhoeae* and *Mycobacterium tuberculosis*, RhierBAPS generated substantially better clustering than PopPUNK, as judged by the Silhouette index. This likely represents the absence of true strains in these species, which are homogeneous compared with the other species studied, and therefore do not exhibit the discontinuous but correlated divergence in π and a assumed in the clustering stage. In *N. gonorrhoeae*, the accessory genome content consists of a small number of prophage, three plasmids and the 80 kb gonococcal genomic island (GGI), all of which can vary independently of core genome divergence (Bennett et al., 2010; Hamilton et al., 2005; Morse et al., 1986). Using the fit based on the network score, we were able to successfully split the population into 132 clusters. Inspection in Microreact revealed a clade composed of a polyphyletic mixture of clusters 5 and 10 (fig S11). Their close relationship within the core genome tree suggested the difference in clustering reflected divergence in their accessory genome. To identify the specific loci responsible for this split, microbial genome-wide association software (pyseer) was applied to these isolates, finding 3,679 k-mer distinguishing the clusters 5 and 10 (Lees, Galardini, et al., 2018; Lees, Vehkala, et al., 2016). The top hits recovered following mapping of these k-mers to reference sequences were the GGI and phage sequences, confirmed as being the distinctive loci through manual inspection.

For such cases where there is independent core and accessory evolution, we therefore also implemented a more suitable model which just uses one of the core or accessory distances, rather than a combined score. All three clusterings can be jointly inspected using the Microreact output (for example `https://microreact.org/project/S1KgTKteQ`). In this particular cluster, the core distances no longer separated the isolates with the GGI and prophage, whereas the accessory distances gave a similar clustering to the combined boundary.

Applying the same analysis to *M. tuberculosis* (`https://microreact.org/project/rJ4lfHtZX`) demonstrated the PopPUNK phylogeny accurately reconstructed the previously-identified lineages in this population (Cohen et al., 2015). While the top-level RhierBAPS effectively identified these lineages, the PopPUNK core genome sequence clusters were much more finely grained, resembling the categorisation into spoligotypes, which are informative for more detailed epidemiology. As PopPUNK's clusters can be easily assigned to lineages or RhierBAPS clusters using the core phylogeny, this ensures the high-resolution links identified using this software can also be used for analysis at broader levels. Increasing the sketch size used for this analysis would also allow for finer differences in π to be measured within spoligotypes, in principle down to single SNP resolution.

Such detailed epidemiology can also be valuable within more diverse species, such as when analysing of individual strains. As an example of such an hierarchical study of within-strain diversity, a PopPUNK analysis of the *S. pneumoniae* PMEN14 lineage (Croucher, Chewapreecha, et al., 2014) was compared to previous accessory genome study using PANINI, which applies t-SNE to the accessory gene presence/absence matrix generated by Roary (Abudahab et al., 2018) (Fig S16). Due to the lower sequence divergence within a single lineage, we compared results using the default, PopPUNK was run with sketch sizes of 10^4 (`https://microreact.org/project/H1UsF5CxX`) and a increased size of 10^5 (`https://microreact.org/project/H1Av59ClQ`). In both cases, the phylogeny and accessory clusterings were similar, with the t-SNE projection recapitulating the main results from the PANINI analysis: group 5, lacking the Tn*916* antibiotic resistance element, were resolved as being separate from the rest of the population, while groups two and three, both carrying two prophage, were separated from the non-lysogenic group one, despite them being polyphyletic in the core genome tree.

2 Supplemental methods

Determining the range k_{min} to k_{max}

As noted by Ondov et al. (2016), the probability p of a random match of a k-mer of length k is:

$$p = \frac{1}{\frac{(\bar{\Sigma})^k}{L} + 1} \tag{1}$$

where L is the total length of the sequence, and Σ is the alphabet. In this case the alphabet are the DNA bases A, C, G and T, so $\bar{\Sigma} = 4$. To find the minimum k-mer length k_{min} which sets a desired maximum probability of random matches p_{random}, eq. (1) can be simply rearranged as follows:

$$k_{\text{min}} = \frac{\log(L) + \log(1 - p_{\text{random}}) - \log(p_{\text{random}})}{\log(4)} \tag{2}$$

k_{max} is set as 29 by default, as we found empirically that this provided enough points to reliably fit each regression while minimising computational burden. This can be increased by the user if desired, up to the maximum k-mer length mash was compiled to support.

Automated classification of within-cluster distances

We implemented two models to classify which distance pairs (π, a) are within the same cluster, the choice of which depends on the dataset being used. The first fits a two-dimensional Gaussian mixture model (2D GMM) to a random subsample of up to 10^5 distance pairs using variational Bayes inference. We use the default implementation in scikit-learn 0.19 with a Dirichlet Process prior on weights, choosing the best final likelihood from five k-means initial starts. The maximum allowed number of mixture components (K) can be specified by the user, depending on the plotted distribution of pairwise π and a distances; by default, K is set to two. We use membership of mixture component closest to the origin (checking that it contains at least one point in the reference database, as the Dirichlet process prior allows mixture component weights to be set ≈ 0) to define within-cluster distances. All distance pairs are then classified with the fitted model.

The alternative approach uses HDBSCAN to classify a subsample of 10^5 points using the Boruvka ball tree algorithm (McInnes et al., 2017). This set of points is iteratively analysed with progressive reductions in both the minimum number of samples required to initiate the search for a cluster, which defines the how conservative the clustering is, and the minimum cluster size, which determines the threshold number of points a cluster must contain, until there are fewer than D clusters (100, by default), and extent of the points in the cluster closest to the origin (assumed to represent within-strain distances) do not overlap with those in the most numerous cluster (assumed to represent between-strain distances) on either axis.

Use of within-cluster distances to define a reference network and clusters

We use networkx v2.1 (Hagberg et al., 2008) to construct an undirected graph with unweighted edges to define population clusters. Each sample in the reference database is a node in this graph, and distances classified as within-cluster by the above model are added as edges between the corresponding nodes. Clusters are then simply defined by extracting the connected components of this network, and ordered by the number of isolates they contain, from largest to smallest. Evaluation of the network structure uses a score, n_s:

$$n_s = \text{transitivity}(1 - \text{density}) \tag{3}$$

This score ranges between zero and one, with values close to the upper bound corresponding to a good fit, as every isolate in a cluster should share a within-cluster link to every other member of

the cluster. This is achieved in spite of the sparseness enforced by the (1 - density) term, which is necessary to subdivide the overall population (Fig S9). After definition of clusters, we randomly select just one member of each clique in the network (sets of nodes where each member node is mutually connected to every other member node) to use in an updated reference database. This removes redundancy in the distances that need to be calculated for database querying, and increases the speed at which further batches of data can be assigned.

Refinement of distance classification using network properties

Both the 2D GMM and HDBSCAN modes rapidly and robustly identified the main clusters of within- and between-strain distances in π-a space, which were typically well-resolved. However, being conservative in assigning points to the within-strain cluster is not intrinsic to these methods, which treat clusters symmetrically. The relatively small numbers of false positive within-strain assignments from the low density of points between clusters strongly impacted upon network structure by linking components, and consequently the strain definitions. Therefore a model refinement mode was developed to precisely delimit the range of π-a distances that were treated as within-strain links, in order to maximise n_s. If a 2D GMM or HDBSCAN model has already been fitted, then we construct a line between the means of the within- and between-strain clusters, then draw a decision boundary normal to this line (Fig 1). If neither model fitted satisfactorily, then the mean positions of the within- and between-strain cluster means can be provided manually.

We then allow this triangular boundary, distinguishing within- and between-strain distances, to be moved over a user-set range forward and backward from the starting point. We globally maximise n_s first by testing the network score when placing the boundary at 40 equally spaced points over the allowed range. Local maximisation near this global optimum is then performed using Brent's method (Brent, 1973). We have also made it possible to run this optimisation with a vertical boundary (using core distances, π, only) or a horizontal boundary (using accessory distances, a, only) if desired.

Defining the cluster of query sequences using a previous reference database

New sequences can be rapidly assigned to either a pre-existing cluster, or start to form a new cluster, by addition to the reference network. First, distances are calculated between each query and each member of the reference database using the variable *k*-mer method as above. New queries are added as nodes in the network, and those distance pairs classified as within-cluster are added as edges. As before we define clusters as the connected components of this network, while ensuring labelling consistency with the reference database. By only calculating reference-query distances rather than all distances, we can perform assignment of M queries using a reference database with N members using NM distance calculations rather than $(N+M)^2$. If the database is being updated for further querying, all M^2 query-query distances are also calculated (for a total of $NM+M^2$ distances) so that cliques in the network can still be used to extract representatives for each cluster. However, in practice the construction of M sketches is the most computationally expensive step, which gives roughly linear query time in both cases.

Analysis of low diversity pathogens

For species with a monomorphic population structure there is not necessarily a clear correlation between a and π. In this case it is logical to define independent sets of clusters using the two distances separately. An example of this is *N. gonorrhoeae*, which we describe fully in Text S1. We used a subset of isolates, all of which were in the same cluster based on π but contained two different clusters based on a. We performed a genome-wide association study to determine the specific genes responsible for the two different a-based clusters using pyseer v1.1.0 to associate *k*-mers counted from the entire π-based cluster, and using the a-based cluster as the phenotype.

Default settings were used, with the kinship matrix generated using the maximum-likelihood tree under a mixed effect association model (Lees, Galardini, et al., 2018). We mapped the significant *k*-mers to two reference genomes, which between them contain all known accessory elements for *N. gonorrhoeae*.

Code optimisation for large datasets

We optimised our code such that datasets with up to $\sim 10^4$ samples could be analysed in a single step. Where possible, we used numba v0.36.2 to compile functions (Lam et al., 2015) and exploited multithreading of Mash sketching and distance calculations. We also multithreaded the regressions to calculate core and accessory distances, using the sharedmem package (v0.3.5) to avoid copying and storing large distance matrices in main memory (Feng et al., 2017). We infer sequence labels of rows in distance matrices by their order, rather than storing them in memory. For larger datasets, fitting a reference model to a subset of samples, then adding in query sequences iteratively makes analysis tractable.

Automated plotting

We automatically produce plots to diagnose dataset characteristics, quality of model fit and assignment of distances to clusters using matplotlib v2.1.2 (Hunter, 2007). For the distances selected for model fitting, we plot contours of a kernel density estimate using an Epanechnikov kernel. This can also be used to identify outliers with contamination; we provide a program to remove these isolates from reference databases. Plots are generated for each model type showing details of its fitted parameters, together with cluster assignment of the distances. For 2D GMMs, we also plot equal likelihood contours and the decision boundary for within- and between-cluster assignments (Fig S7).

3 Supplemental tables

Table S1: Parameters used in BacMeta simulations (Sipola et al., 2018). Only a single population was simulated (NPOP = 1), hence the migration rate was set to zero. All parameters not specified here were the default values used by the software.

Parameter code	Parameter description	Parameter value	Default when other parameters vary
GENR	Number of generations	25000	25000
NBAC	Number of individuals in population	1000	1000
SEQS	Proportion of individuals sampled for analysis	0.025	0.025
LOLE	Length of individual loci	1000	1000
NLOC	Number of loci in each individual	100	100
MUTR	Point mutation rate	1E-7, 5E-7, 1E-6, 5E-6, 1E-5	5E-6
INSR & DELR	Indel mutation rate	0.01, 0.02, 0.03, 0.04, 0.05	0 or 0.025
RECR	Recombination rate	0, 0.05, 0.5, 1, 2	-
INSL & DELL	Indel length parameter	100	100
RECA	Recombination acceptance parameter for similarity test	0	0
MIGR	Migration probability	0	0

Table S2: Resource and result comparison for each dataset. For all methods we quote the total CPU time used, and maximum memory. Both PopPUNK and RhierBAPS use multithreading with close to 100% efficiency, so total wall-time was lower than these estimates roughly by a factor of the number of cores used.

Species	Samples	Publication reference	PopPUNK microreact	PopPUNK/ RhierBAPS microreact	Resource use (single thread)		
					Roary	RhierBAPS	PopPUNK
Staphylococcus aureus	284	Aanensen et al. (2016)	`https://microreact.org/project/HJJEpu1Rf`	`https://microreact.org/project/rJCRZFx0M`	11.2 hrs CPU<br>3.6 GB RAM	6.3 hrs CPU<br>4.9 GB RAM	0.6 hrs CPU<br>0.3 GB RAM
Escherichia coli	1508	Kallonen et al. (2017)	`https://microreact.org/project/B1tM9YyAM`	`https://microreact.org/project/B1kLl9yAG`	144 hrs CPU<br>36.2 GB RAM	1662 hrs CPU<br>44.7 GB RAM	22.2 hrs CPU<br>0.8 GB RAM
Salmonella enterica	847	Alikhan et al. (2018)	`https://microreact.org/project/Skg0j9sjz`	`https://microreact.org/project/Sk7brUBWX`	101 hrs CPU<br>21.0 GB RAM	24.4 hrs CPU<br>142 GB RAM	2.6 hrs CPU<br>0.5 GB RAM
Listeria monocytogenes	128	Kremer et al. (2017)	`https://microreact.org/project/S1ktRPJCM`	`https://microreact.org/project/r1gDQcyRf`	30.7 hrs CPU<br>1.9 GB RAM	27.2 hrs CPU<br>7.8 GB RAM	0.2 hrs CPU<br>0.2 GB RAM
Haemophilus influenzae	75	Koelman et al. (2017)	`https://microreact.org/project/BJ_se01CM`	`https://microreact.org/project/HyUhLte0G`	21.2 hrs CPU<br>1.0 GB RAM	11.2 hrs CPU<br>5.2 GB RAM	0.1 hrs CPU<br>0.2 GB RAM
Neisseria meningitidis	882	Lees, Kremer, et al. (2017)	`https://microreact.org/project/H1ZgUY1Af`	`https://microreact.org/project/SkZe9tlAM`	17.2 hrs CPU<br>8.1 GB RAM	193 hrs CPU<br>37.0 GB RAM	2.8 hrs CPU<br>0.5 GB RAM
Neisseria gonorrhoeae	1102	Grad et al. (2016)	`https://microreact.org/project/BkIhiwSx7`	`https://microreact.org/project/S1KgTKteQ`	26.2 hrs CPU<br>10.9 GB RAM	239 hrs CPU<br>21.0 GB RAM	2.6 hrs CPU<br>0.6 GB RAM
Streptococcus pyogenes	675	Lees, Vehkala, et al. (2016)	`https://microreact.org/project/BJNtbheCf`	`https://microreact.org/project/SJoAae6Mm`	9.3 hrs CPU<br>5.3 GB RAM	323 hrs CPU<br>39.3 GB RAM	0.7 hrs CPU<br>0.3 GB RAM
Streptococcus pneumoniae	616	Croucher, Finkelstein, et al. (2013)	`https://microreact.org/project/SJxxLMcaf`	`https://microreact.org/project/ByIBsu--X`	9.2 hrs CPU<br>5.8 GB RAM	32.6 hrs CPU<br>9.0 GB RAM	0.7 hrs CPU<br>0.3 GB RAM
Mycobacterium tuberculosis	219	Cohen et al. (2015)	`https://microreact.org/project/HJMChNF-X`	`https://microreact.org/project/rJ4lfHtZX`	19.7 hrs CPU<br>5.0 GB RAM	4.1 hrs CPU<br>4.2 GB RAM	0.4 hrs CPU<br>0.6 GB RAM

Table S3: PopPUNK results for iteratively adding *E. coli* data by year. For each year going forwards, a Microreact instance shows the PopPUNK output using all genomes ('full') and using cliques to choose representatives ('reference') at each stage. The Rand index (Rand, 1971) and adjusted Rand index (Hubert et al., 1985) between these clusters are also reported, which vary between 0 (totally discordant cluster assignment) and 1 (identical cluster assignment).

Surveillance period	Microreact for full analysis	Microreact for reference analysis	Rand index	Adjusted Rand index
2001	https://microreact.org/project/Hycf66PtX	https://microreact.org/project/S1fFrpjYm	1.0	1.0
2001-2002	https://microreact.org/project/B1lru2avKm	https://microreact.org/project/ry0HB6iYQ	1.0	1.0
2001-2003	https://microreact.org/project/BkMQ26wYm	https://microreact.org/project/rybmSpoYQ	1.0	1.0
2001-2004	https://microreact.org/project/Sy23opwYm	https://microreact.org/project/S16krajYQ	0.99983	0.99895
2001-2005	https://microreact.org/project/r1fIs6DY7	https://microreact.org/project/SyE5E6jFX	1.0	1.0
2001-2006	https://microreact.org/project/Sk5hq6DYQ	https://microreact.org/project/SJmZ46sYm	0.99946	0.99620
2001-2007	https://microreact.org/project/ByfYqavt7	https://microreact.org/project/H1wA7poFm	1.0	1.0
2001-2008	https://microreact.org/project/B1Z_tTPKQ	https://microreact.org/project/SJBt7TjFX	1.0	1.0
2001-2009	https://microreact.org/project/Hkhy9TvtX	https://microreact.org/project/B1eV7aitX	0.99939	0.99541
2001-2010	https://microreact.org/project/Hkiju6wKX	https://microreact.org/project/SJ0gQastQ	1.0	1.0
2001-2011	https://microreact.org/project/rJGAHaPtm	https://microreact.org/project/BkX-3ojFm	0.99919	0.99573

Table S4: The average within-cluster and between-cluster SNP distances for each species, for both the PopPUNK and RhierBAPS cluster assignments (first level; for *L. monocytogenes* second level). These distributions are also plotted in Fig S12.

Species	Samples	PopPUNK clusters		RhierBAPS clusters	
		Average within cluster distance	Average between cluster distance	Average within cluster distance	Average between cluster distance
Staphylococcus aureus	284	136	10041	991	10286
Escherichia coli	1508	1193	30527	1880	30636
Salmonella enterica	847	10257	67688	10298	67703
Listeria monocytogenes	128	64	54214	1716	54852
Haemophilus influenzae	75	1356	30145	13208	31323
Neisseria meningitidis	882	3279	18726	3134	17681
Neisseria gonorrhoeae	1102	319	2843	510	2863
Streptococcus pyogenes	675	91	6877	5725	7020
Streptococcus pneumoniae	616	308	1864	770	1873
Mycobacterium tuberculosis	219	20	809	141	896

Table S5: Proportion of pairs in each assigned cluster containing polyphyletic cluster assignments, excluding singleton clusters. For both the PopPUNK and RhierBAPS (first level) clusters for each species we show the proportion of total pairs with a polyphyletic assignment, and also the number of clusters containing at least one polyphyletic assignment. In *M. tuberculosis* splitting the dataset into mostly singleton clusters prevents polyphyly, meaning only a small proportion of the population were included in the test presented here.

Species	Samples	PopPUNK clusters		RhierBAPS clusters	
		Proportion of total polyphyletic pairs	Number of clusters containing a polyphyletic pair	Proportion of total polyphyletic pairs	Number of clusters containing a polyphyletic pair
Staphylococcus aureus	284	0.000	0/18	0.069	1/9
Escherichia coli	1508	0.006	1/57	0.013	3/24
Salmonella enterica	847	0.000	0/11	0.000	0/10
Listeria monocytogenes	128	0.000	0/21	0.051	2/18
Haemophilus influenzae	75	0.000	0/17	0.261	2/7
Neisseria meningitidis	882	0.002	1/32	0.054	4/15
Neisseria gonorrhoeae	1102	0.148	6/46	0.091	2/15
Streptococcus pyogenes	675	0.000	0/109	0.786	3/7
Streptococcus pneumoniae	616	0.000	0/47	0.228	1/19
Mycobacterium tuberculosis	219	0.000	0/14	0.000	0/7

Table S6: Clusters defined by varying number of allele differences in the MLST (7 genes) and cgMLST (1701 gene) scheme for *L. monocytogenes*. For each cutoff (which were chosen to be logarithmically spaced, spanning the PopPUNK defined cutoff) clusters were defined using PopPUNK's network structure. Edges between samples with the stated number of allele differences or fewer were retained. Zero differences therefore corresponds to using ST as clusters. For each assignment the same evaluation metrics as table 1 were calculated. Assigning MLST from reads required 3.1 hrs CPU and 2.0 Gb RAM; assigning cgMLST from assemblies required 1.6 hrs CPU; 0.8 Gb RAM. Using PopPUNK required 0.2 hrs CPU; 0.2 Gb RAM and found 31 clusters which had an average silhouette index of 0.60.

Scheme	Number of clusters	Average silhouette index	Adjusted Rand index (to PopPUNK clusters)
MLST 0-allele (ST)	36	0.31	0.939
MLST 1-allele	31	0.57	0.982
MLST 3-allele	15	0.33	0.644
cgMLST 0-allele (cgST)	128	NA	0.000
cgMLST 1-allele	128	NA	0.000
cgMLST 3-allele	123	-0.02	0.021
cgMLST 10-allele	110	0.01	0.156
cgMLST 30-allele	75	0.12	0.750
cgMLST 100-allele	35	0.50	0.974

Table S7: Clusters defined by varying number of allele differences in the MLST (7 genes) and cgMLST (2360 genes) scheme for *E. coli*. For each cutoff (which were chosen to be logarithmically spaced, spanning the PopPUNK defined cutoff) clusters were defined using PopPUNK's network structure. Edges between samples with the stated number of allele differences or fewer were retained. Zero differences therefore corresponds to using ST as clusters. For each assignment the same evaluation metrics as table 1 were calculated. Assigning MLST from reads required 32.4hrs CPU and 2.4Gb RAM; assigning cgMLST from assemblies required 35.9 hrs CPU; 1.0 Gb RAM. Using PopPUNK required 22.2 hrs CPU; 0.8 Gb RAM and found 130 clusters which had an average silhouette index of 0.40.

Scheme	Number of clusters	Average silhouette index	Adjusted Rand index (to PopPUNK clusters)
MLST 0-allele (ST)	226	-0.10	0.917
MLST 1-allele	131	0.35	0.990
MLST 3-allele	42	0.23	0.565
cgMLST 0-allele (cgST)	1508	NA	0.000
cgMLST 1-allele	1508	NA	0.000
cgMLST 3-allele	1506	0.00	0.000
cgMLST 10-allele	1466	0.02	0.001
cgMLST 30-allele	1189	0.01	0.160
cgMLST 100-allele	539	0.17	0.369
cgMLST 300-allele	216	0.34	0.947
cgMLST 1000-allele	115	0.41	0.997

Table S8: List of Microreact instances produced, by species and purpose. Additionally, files with inferred clusters, phylogenies and t-SNE projections can be downloaded using each link.

Species	Purpose	Microreact instance
Staphylococcus aureus	PopPUNK clusters	`https://microreact.org/project/HJJEpu1Rf`
	Comparison of RhierBAPS and PopPUNK clusters	`https://microreact.org/project/rJCRZFx0M`
Escherichia coli	PopPUNK clusters	`https://microreact.org/project/B1tM9YyAM`
	Comparison of RhierBAPS and PopPUNK clusters	`https://microreact.org/project/B1kLl9yAG`
Salmonella enterica	PopPUNK clusters (with added metadata – used in online tutorial)	`https://microreact.org/project/Skg0j9sjz`
	Comparison of R-hierBAPS and PopPUNK clusters	`https://microreact.org/project/Sk7brUBWX`
Listeria monocytogenes	PopPUNK clusters	`https://microreact.org/project/S1ktRPJCM`
	Comparison of R-hierBAPS and PopPUNK clusters	`https://microreact.org/project/r1gDQcyRf`
Haemophilus influenzae	PopPUNK clusters	`https://microreact.org/project/BJ_se01CM`
	Comparison of R-hierBAPS and PopPUNK clusters	`https://microreact.org/project/HyUhLte0G`
Neisseria meningitidis	PopPUNK clusters	`https://microreact.org/project/H1ZgUY1Af`
	Comparison of R-hierBAPS and PopPUNK clusters	`https://microreact.org/project/SkZe9tlAM`
Neisseria gonorrhoeae	PopPUNK clusters	`https://microreact.org/project/BkIhiwSx7`
	Comparison of R-hierBAPS and PopPUNK clusters	`https://microreact.org/project/S1KgTKteQ`
Streptococcus pyogenes	PopPUNK clusters	`https://microreact.org/project/BJNtbheCf`
	Comparison of R-hierBAPS and PopPUNK clusters	`https://microreact.org/project/SJoAae6Mm`
Streptococcus pneumoniae	PopPUNK clusters	`https://microreact.org/project/SJxxLMcaf`
	Comparison of R-hierBAPS and PopPUNK clusters	`https://microreact.org/project/ByIBsu--X`
	Expanding a species-wide clustering through query assignment (adding another species-wide sample)	`https://microreact.org/project/SkZ23iPbX`
	Expanding a species-wide clustering through query assignment (adding a multidrug-resistant lineage)	`https://microreact.org/project/BkNqKdPb7`
	Expanding a species-wide clustering through query assignment to reference sequences (adding an multidrug-resistant outbreak lineage)	`https://microreact.org/project/Hk-_FOoWX`
	PopPUNK clustering of a single lineage with sketch size 10^4	`https://microreact.org/project/H1UsF5CxX`
	PopPUNK clustering of a single lineage with sketch size 10^5	`https://microreact.org/project/H1Av59ClQ`
Mycobacterium tuberculosis	PopPUNK clusters	`https://microreact.org/project/HJMChNF-X`
	Comparison of R-hierBAPS and PopPUNK clusters	`https://microreact.org/project/rJ4lfHtZX`

4 Supplemental figures

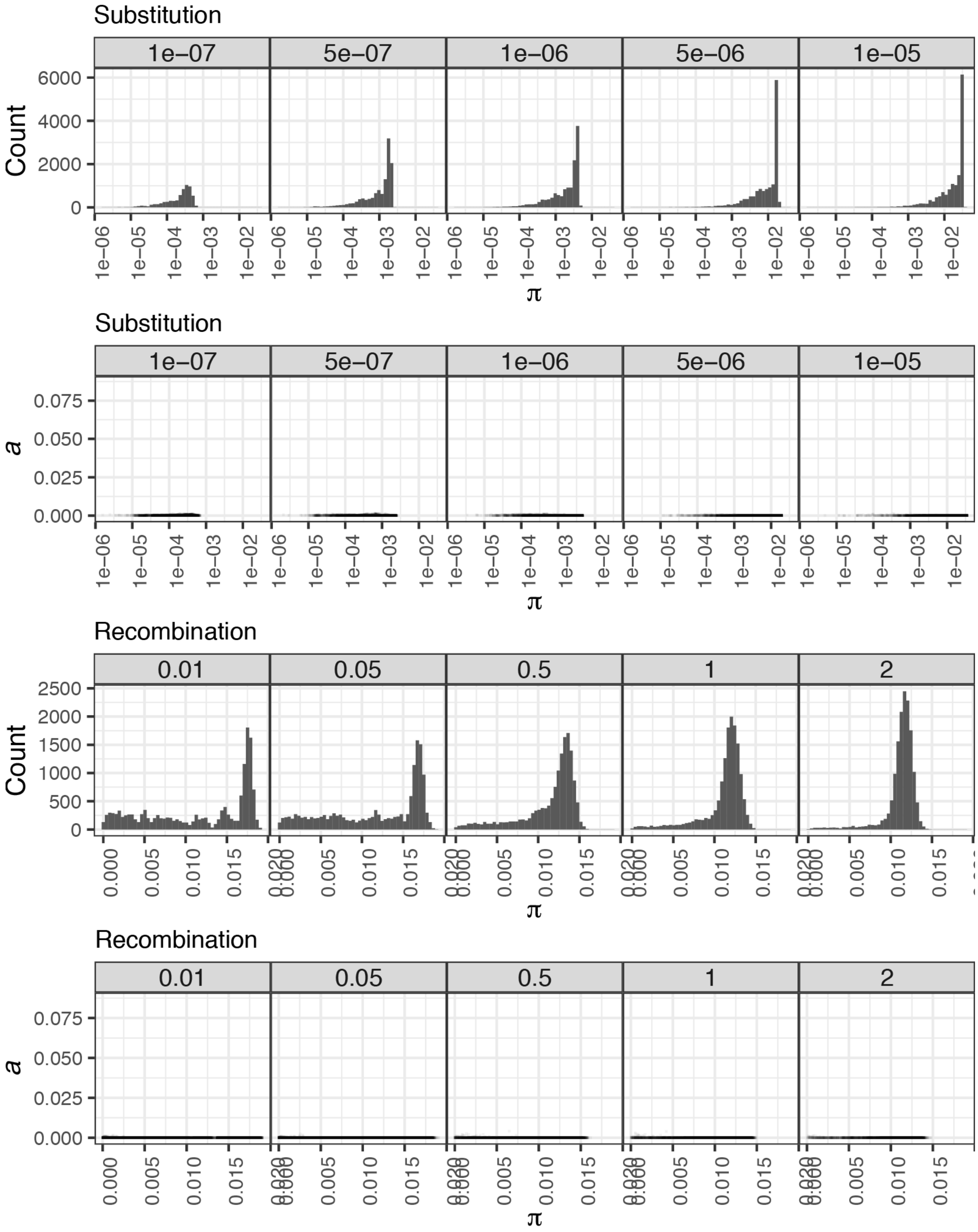

Fig. S1: Comparison between simulated rates of divergence and estimates of diversity by PopPUNK. The histograms show the distribution of pairwise π distances measured by PopPUNK when recombining bacteria diverge through point mutation, occurring at a fixed rate of 5×10^{-6} base^{-1} generation^{-1}. The scatterplots show the distribution of both π and a pairwise distances. These demonstrate recombination affects the range, and pattern, of pairwise distances, as shown in Fig 1C. However, no substantial accessory genome divergence is inferred, demonstrating PopPUNK's specificity in identifying core genome divergence, despite varying levels of sequence exchange between bacteria.

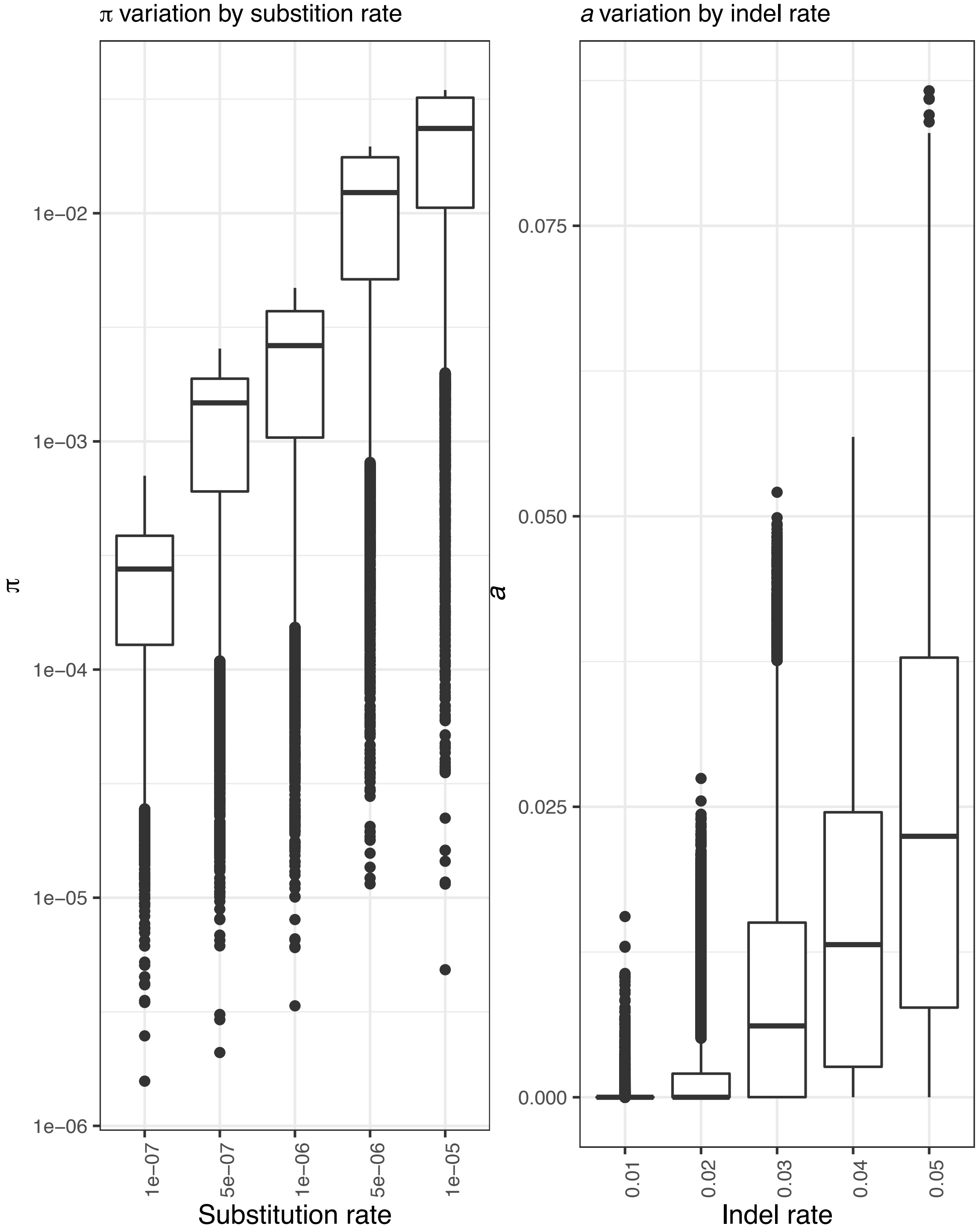

Fig. S2: Comparison between simulated rates of divergence and estimates of diversity by PopPUNK. Left: The distributions of pairwise π distances calculated from simulated bacterial populations are shown on a logarithmic scale relative to the point mutation rate parameter (corresponding to the graphs in Fig 2A). This demonstrates PopPUNK's ability to accurately estimate the density of base substitutions in a genomic dataset across multiple orders of magnitude. Right: The distribution of pairwise a distances calculated from simulated bacterial populations in which the insertion/deletion (indel) rate was varied (corresponding to the graphs in Fig 2B). This demonstrates PopPUNK's ability to estimate differences in sequence content over a range relevant to bacterial populations (Fig 4 and S8).

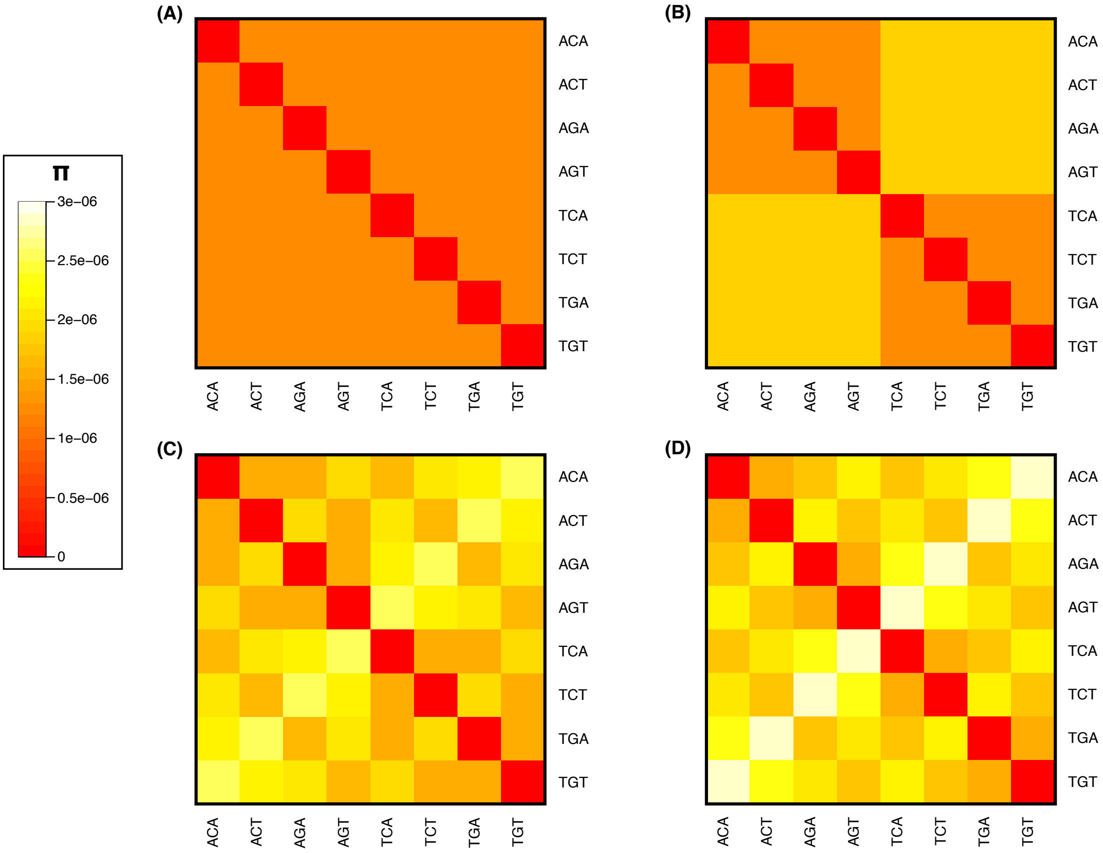

Fig. S3: Effect of sketch size on the precision of PopPUNK distance estimates. Each heatmap represents a pairwise distance matrix between eight different variants of *S. pneumoniae* ATCC 700669, representing all possible combinations at three biallelic SNP sites. The genotypes correspond to the string of bases annotating each row and column. Each cell is coloured according to the indicated pairwise π distance, calculated using PopPUNK with $k_{\text{min}} = 13$ and sketch sizes of (A) 10^3, (B) 10^4, (C) 10^5, and (D) 10^6. Self-comparisons were not computed, but were assumed to be zero. Only the larger two sketch sizes provide sufficient resolution to distinguish all genotypes, indicating these distances are sufficiently precise to detect individual point mutations.

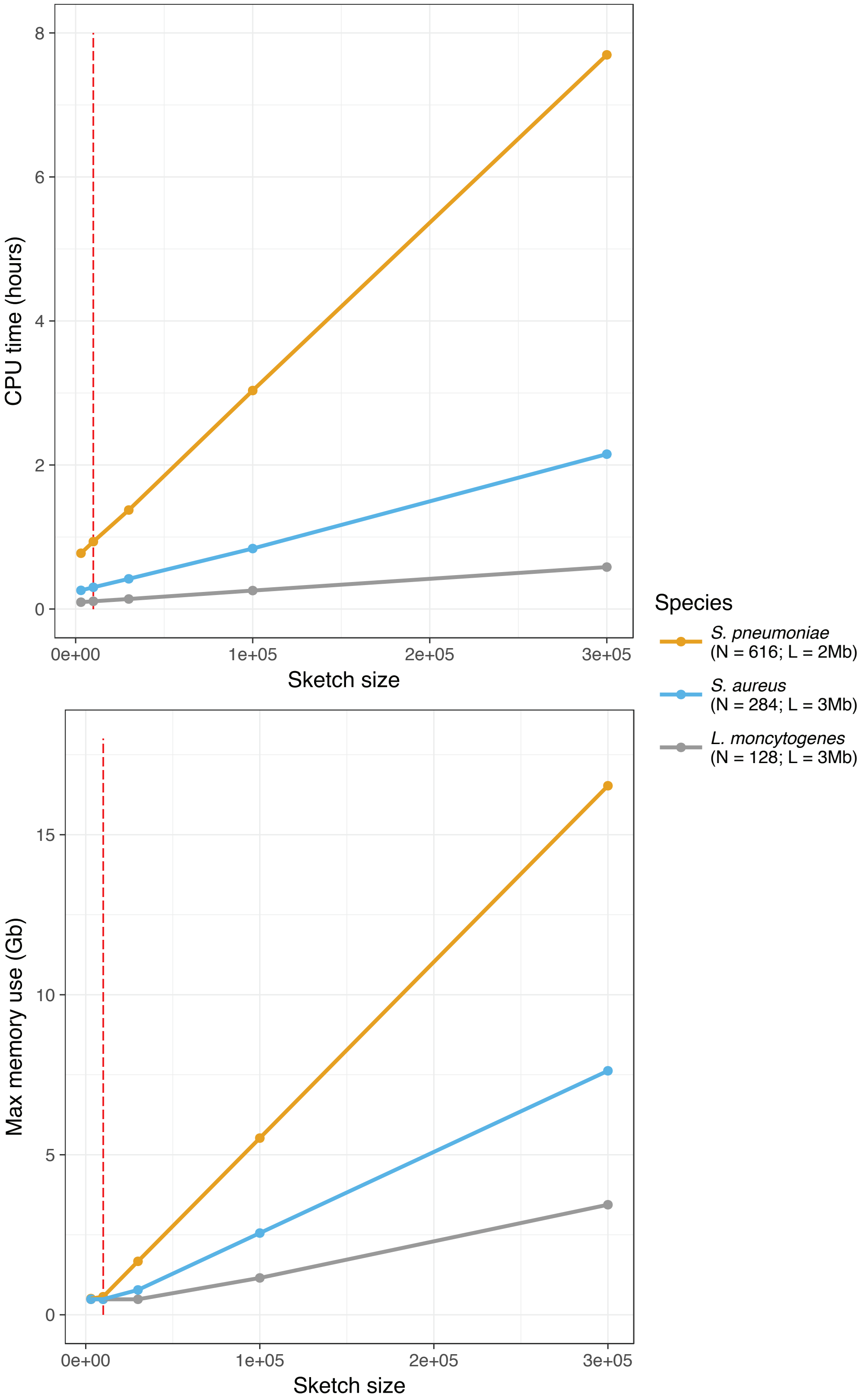

Fig. S4: Resource use with increasing sketch size on three datasets: *S. pneumoniae* (gold), *S. aureus* (blue), *L. monocytogenes* (gray) each with N samples and average genome length L. The x-axis is the mash sketch size, with the default 10^4 indicated by a dashed red line. Top: Single core CPU use in hours. Bottom: Maximum memory use in Gb, when running eight threads.

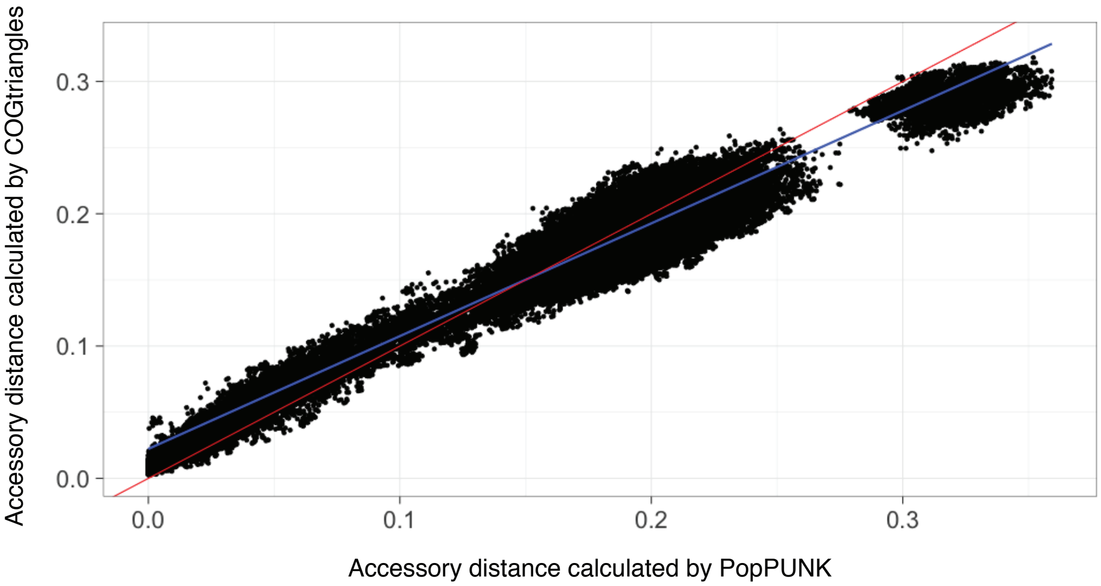

Fig. S5: Comparison of pairwise accessory distances, *a*, calculated for the Massachusetts *S. pneumoniae* population using both PopPUNK and a Glimmer3 and COGtriangles-based approach, as described in Croucher, Finkelstein, et al. (2013). These estimates lie close to the line of identity, unlike those calculated from Roary, shown in Fig 4. The adjusted R^2 is 0.91.

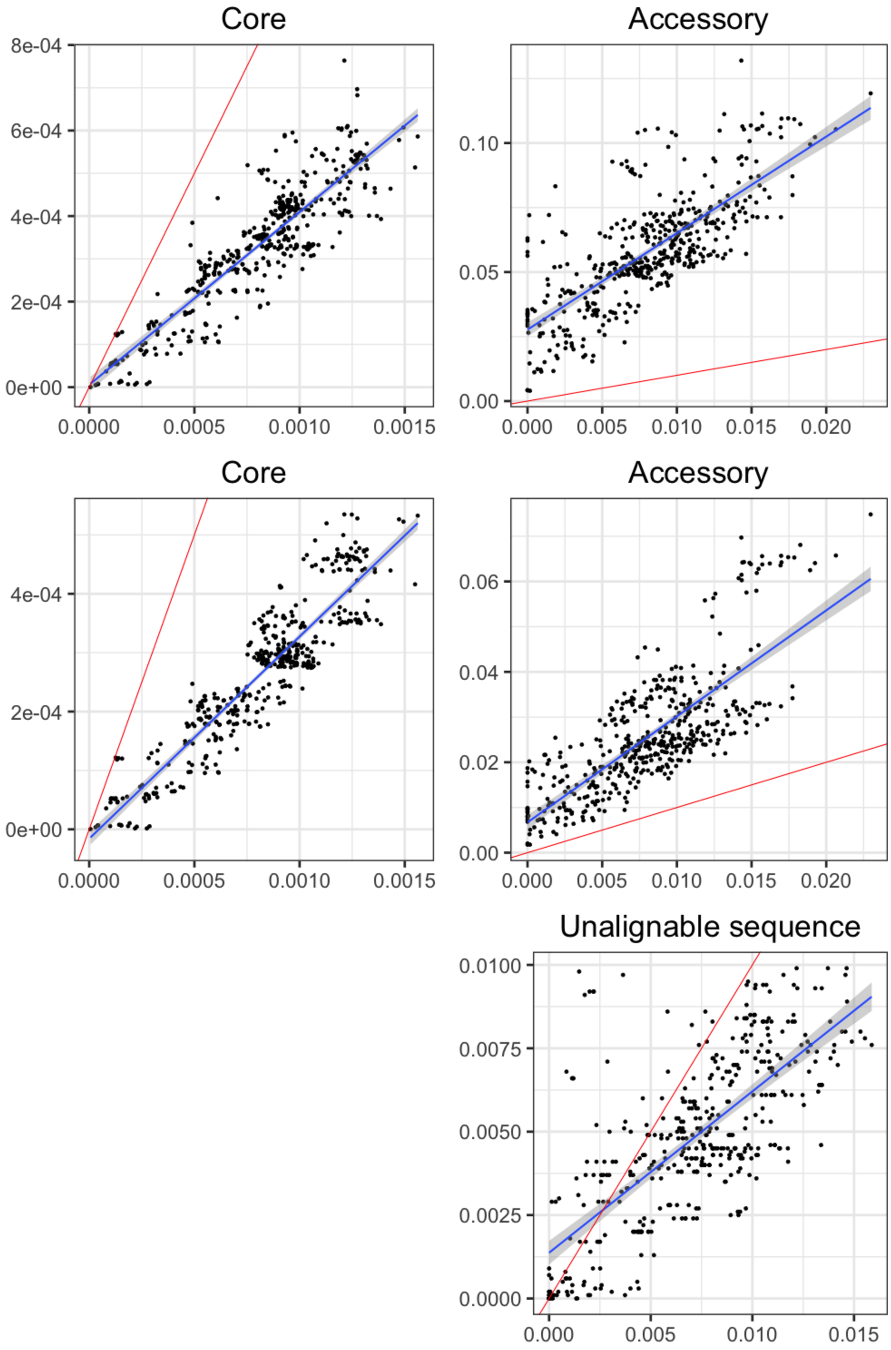

Fig. S6: Analysis of complete *M. tuberculosis* genomes. Thirty-three complete *M. tuberculosis* genomes available from the ENA were analysed using Roary and PopPUNK, and the divergence in the (A) core and (B) accessory genomes were plotted as in Fig 3. This shows the same discrepancies between the methods is observed as with the draft genome collection, indicating the differences are not due to the difficulties in assembling the repetitive loci within these genomes. The Roary analysis was re-run without using synteny to define orthologues (option '-s'), and the results compared to the PopPUNK analysis in (C) and (D). This demonstrates the discrepancy between the accessory distance estimates are substantially reduced, suggesting much of the accessory variation inferred by Roary relates to the difficulty of identifying orthologues, rather than genuine differences in sequence content, which are rare in *M. tuberculosis*. (E) The third row contains one panel, comparing the PopPUNK accessory divergence estimates with the proportion of each genome found to be unalignable in pairwise comparisons with nucmer, using default settings. These methods exhibit a much closer agreement in the proportion of divergent sequence in *M. tuberculosis*.

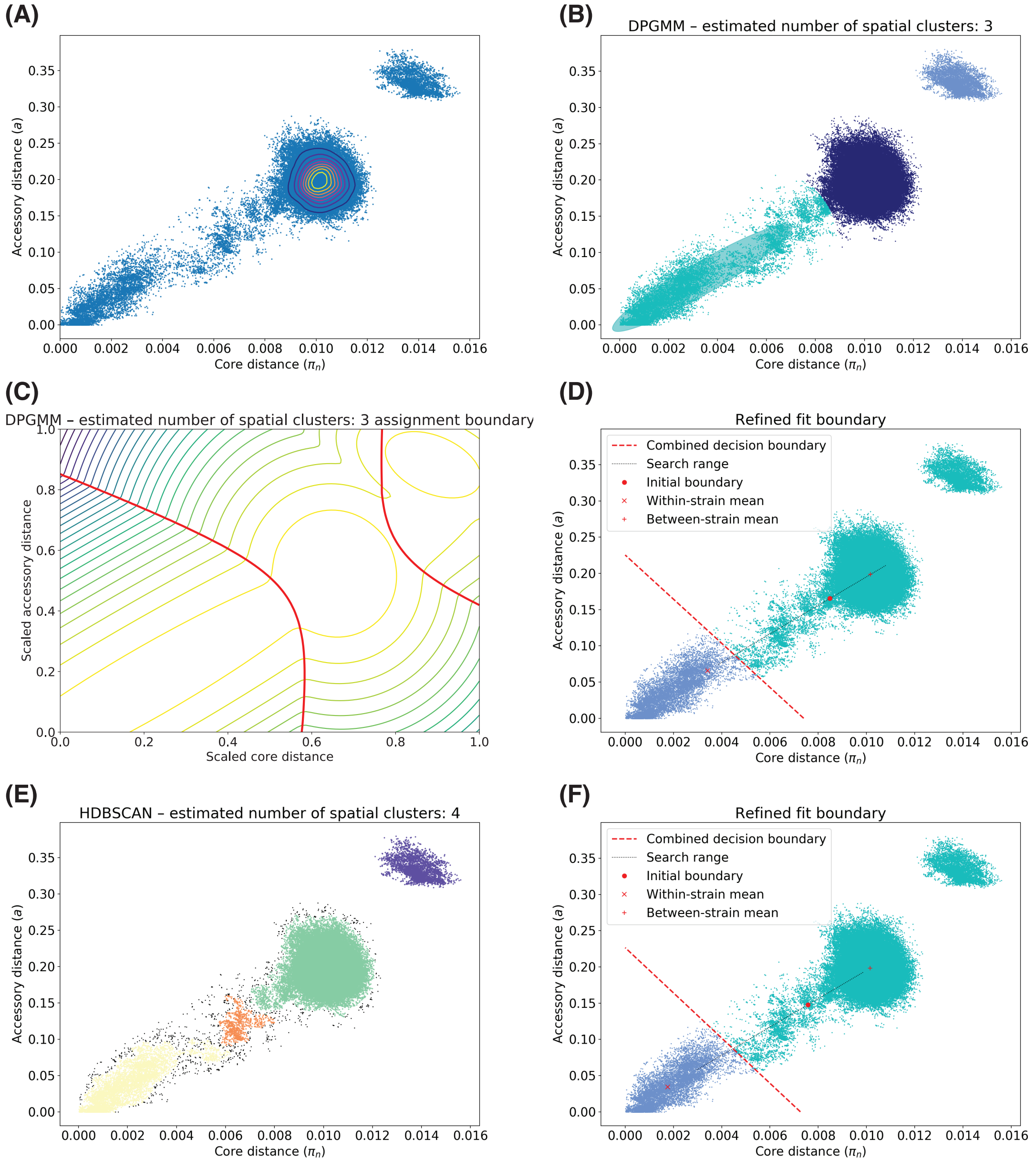

Fig. S7: Example of the output from the model fitting to the *S. pneumoniae* dataset. Plots A, B, D, E, F have π on the x-axis and a distance on the y-axis; plot C has these distances linearly scaled between zero and one. (A) The distribution of all pairwise distances, with equal-density contours from a kernel density estimate. (B) A 2D GMM fit, with three components. (C) Equal likelihood contours of the fit in panel (B), with the red lines marking the decision boundaries between 2D Gaussian distribution components. (D) Refinement of the fit in panel (C), with the final boundary in red and the search space a dashed black line. e) The HDBSCAN fit. The number of clusters is automatically estimated as four. Black points are 'noise-points' not assigned to any cluster. (F) Refinement of the fit in panel (E). A different search range was set than in panel (D), but in both cases the correct global optimum is found.

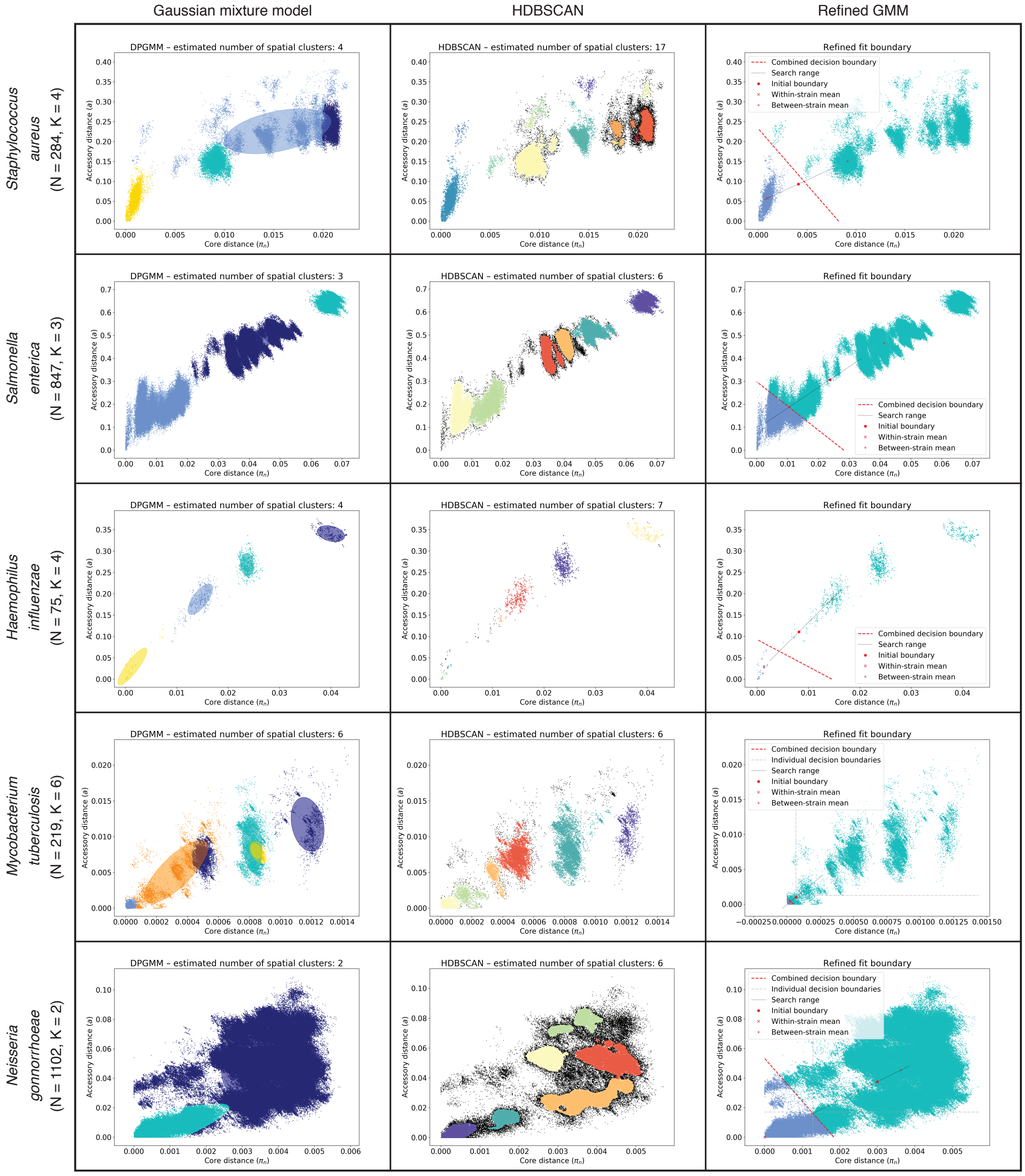

Fig. S8: PopPUNK model fitting output for species listed in Table 1 but not shown in Fig 4. Each row is a species, with each plot showing the distribution of core (π) and accessory (a) distances and points coloured by their predicted cluster. The cluster closest to the origin is the within-strain cluster. The 2D GMM fits are shown in the left column, which also shows ellipses representing the mean and covariance of the fitted mixture components. The HDBSCAN plot in the centre column shows unclassified noise points as black. The final column shows the decision boundaries calculated by maximising the network score to refine the 2D GMM fit. *Staphylococcus aureus* and *Salmonella enterica* have similar π-a distribution properties to *Escherichia coli*. *Haemophilus influenzae* has similar deep branching structure to *Listeria monocytogenes*, and good quality clustering is obtained even with a small number of isolates.

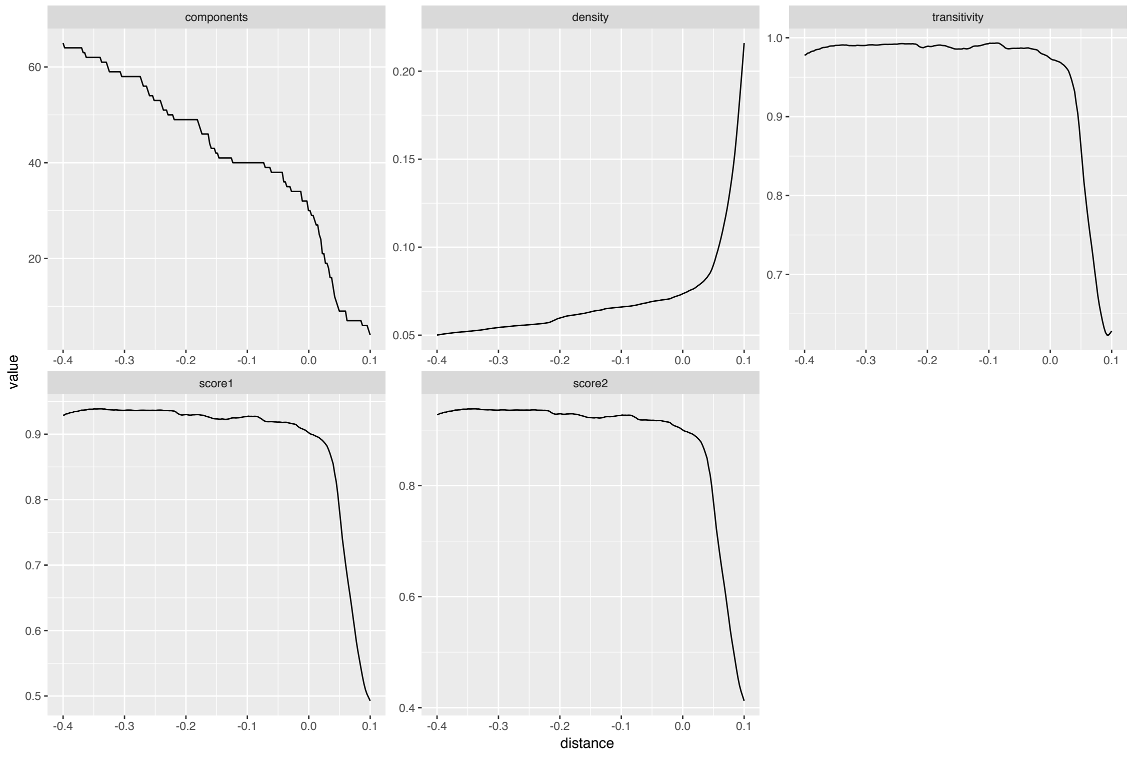

Fig. S9: Plot of number of clusters, network density, transitivity and two score functions as a function of moving boundary position in refine fit mode, on the *S. pneumoniae* dataset. We tried two possible score functions: score 1 = transitivity * (1 - density); score 2 = transitivity - density. We used score 1 throughout, due to its more intuitive scaling.

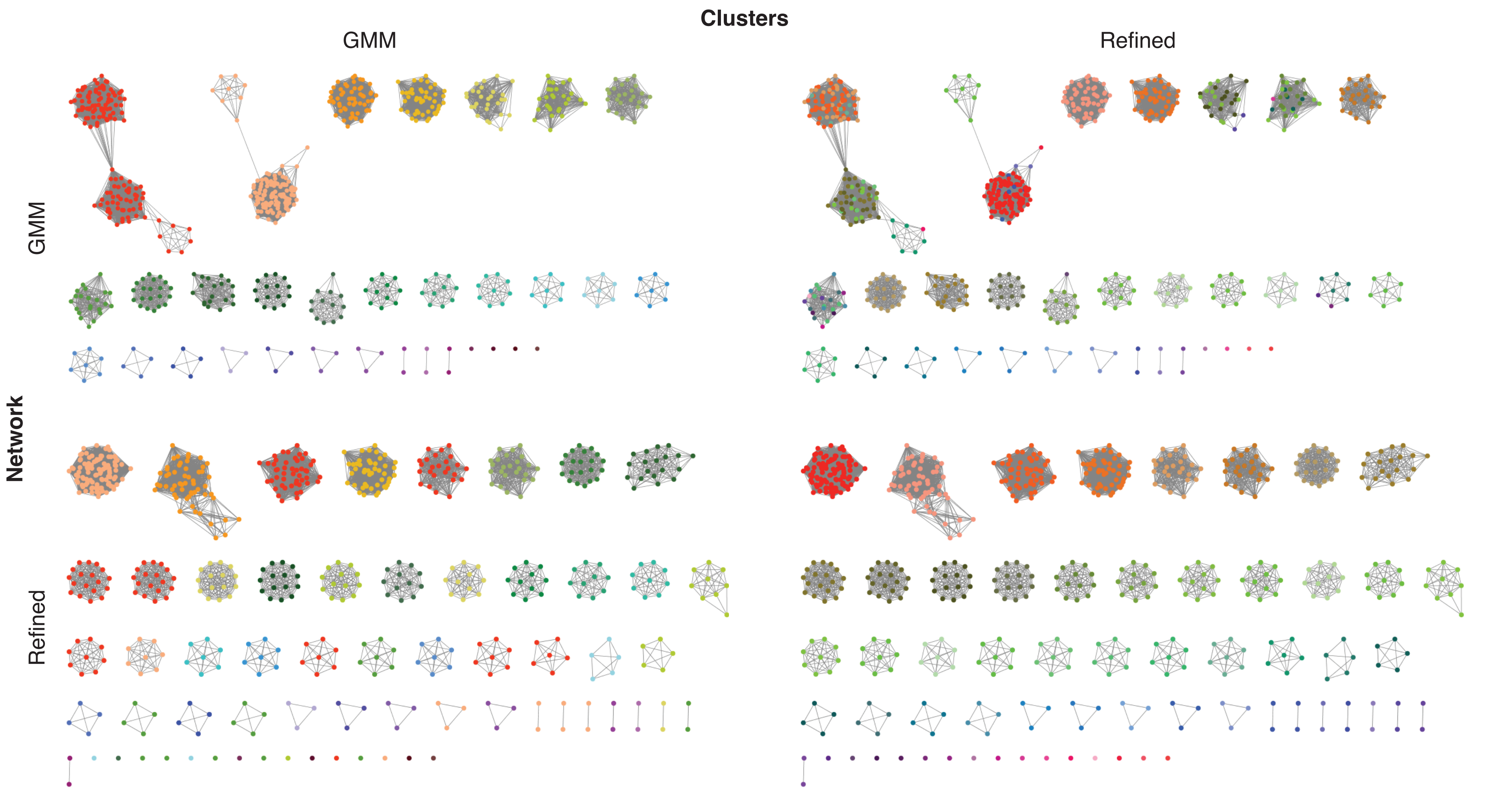

Fig. S10: Effects of refining strain distance threshold on network structure. The networks shown represent the of 616 *S. pneumoniae* genomes from Massachusetts, as shown in Fig 5A and B. The top row shows the network structure emerging when edges were generated for all pairwise distances identified as being within-strain by the GMM fit shown in Fig 4 and Fig S7. The bottom row shows the corresponding network following refinement of the threshold by optimising the network score n_s. This structure contains stronger clustering, with fewer sparse links between components. The left column is coloured according to the GMM clustering, showing the distinct components in the refined network that share colours due to likely spurious links in the GMM network. The right column is coloured according to the refined clustering, with components containing a mixture of colours in the GMM network representing groupings that were disaggregated once a more stringent restriction on within-strain links was imposed.

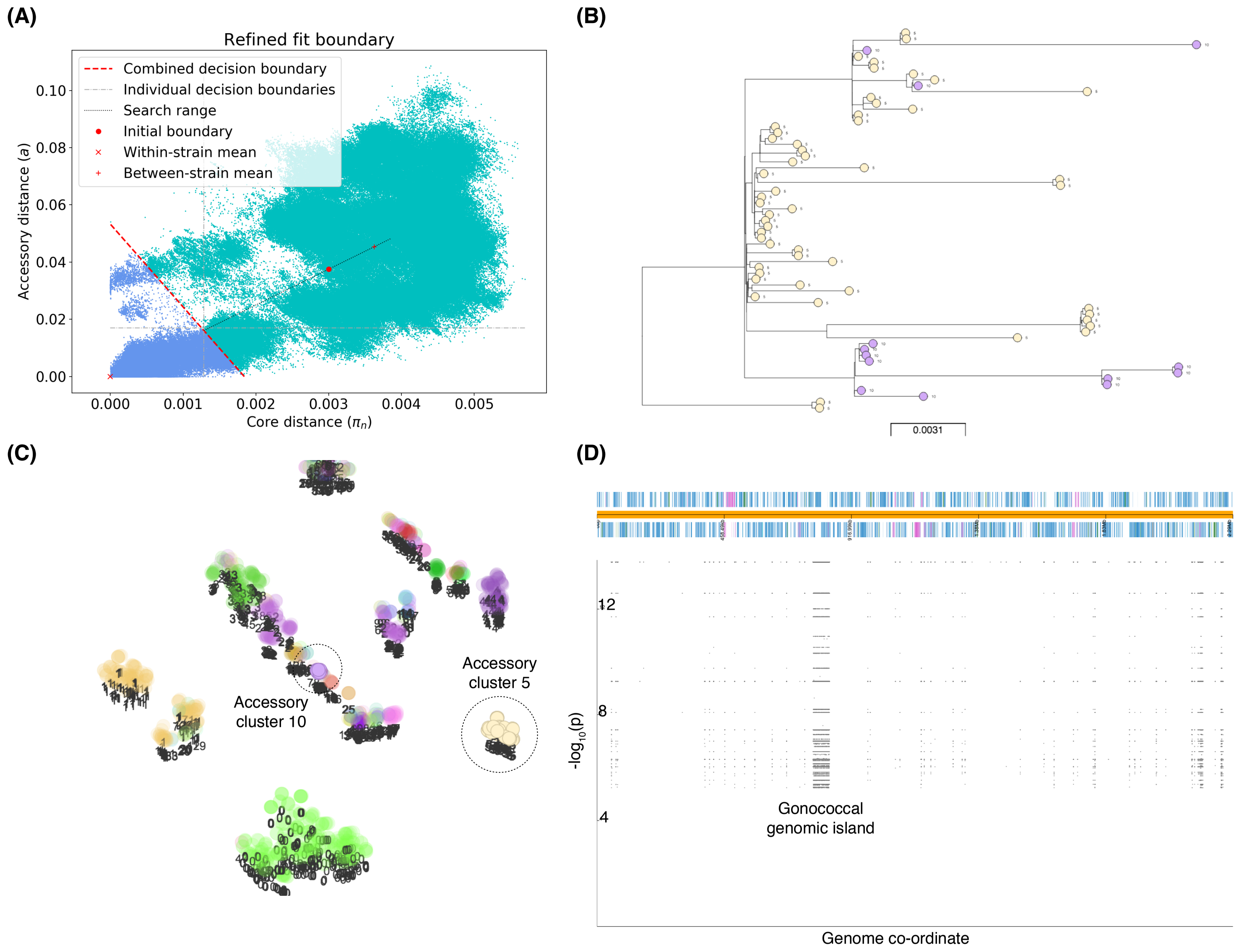

Fig. S11: PopPUNK analysis of *Neisseria gonorrhoeae*. (A) Result of optimising the position of the decision boundary on the combined distances (red dashed line), as well as core and accessory only (dashed gray lines). These methods resulted in 132, 114 and 92 clusters respectively. (B) Example of a single core cluster, which is split into separate clusters using either the combined or accessory boundary. The phylogeny tips are coloured by combined cluster. (C) Accessory t-SNE projection, with the clusters in panel B highlighted, confirming they diverge. (D) Manhattan plot of *k*-mers associated with the subcluster split in panel B, which show the GGI is primarily responsible for this difference.

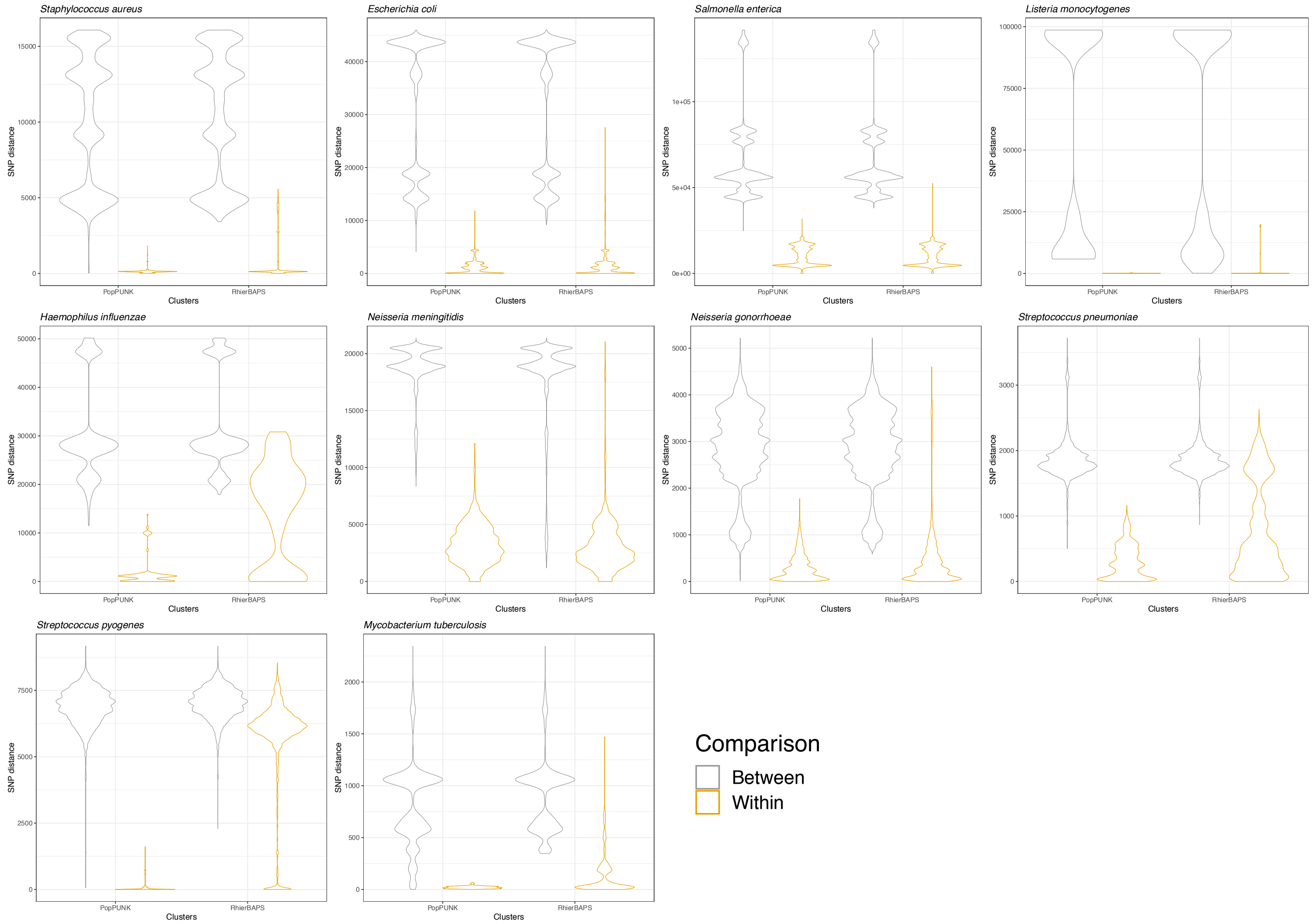

Fig. S12: The distributions of within-cluster and between-cluster SNP distances for each species, for both the PopPUNK and RhierBAPS cluster assignments (first level; for *L. monocytogenes* second level). Grey shows SNP distances between different clusters; gold distances within the same cluster. The means are listed in table S4.

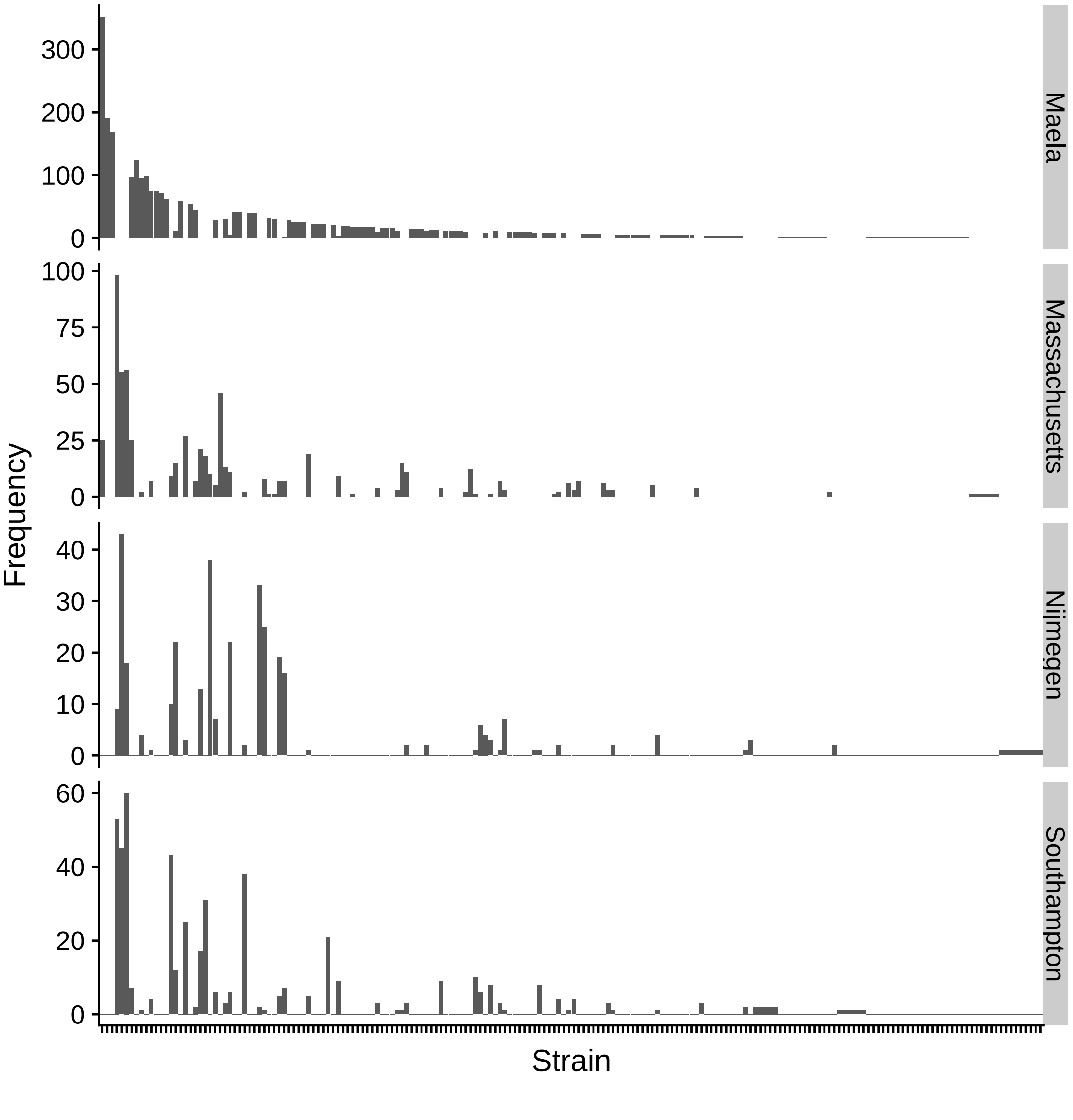

Fig. S13: For each of the four *S. pneumoniae* populations defined in Corander et al. (2017), a bar is shown with the count of samples in each strain defined by PopPUNK in the overall cluster assignment. Clusters are ordered on the x-axis in decreasing order of frequency in the entire dataset, with the same labels for each population.

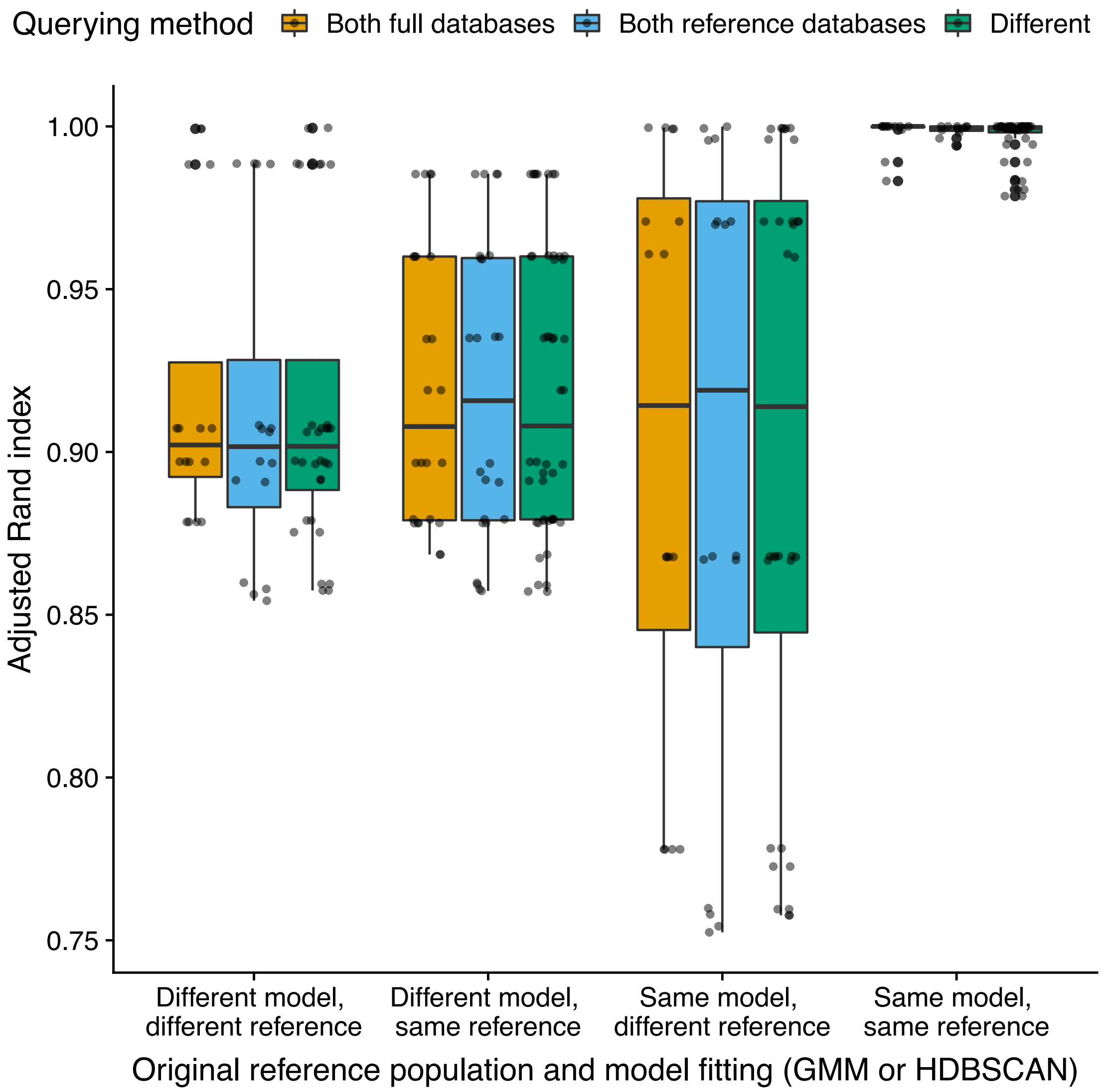

Fig. S14: Boxplots showing the same comparisons as in Fig 5C, but with similarity between clusterings quantified as the adjusted Rand index. This shows the consistency of the strains identified by PopPUNK does not represent chance overlap between the clusterings.

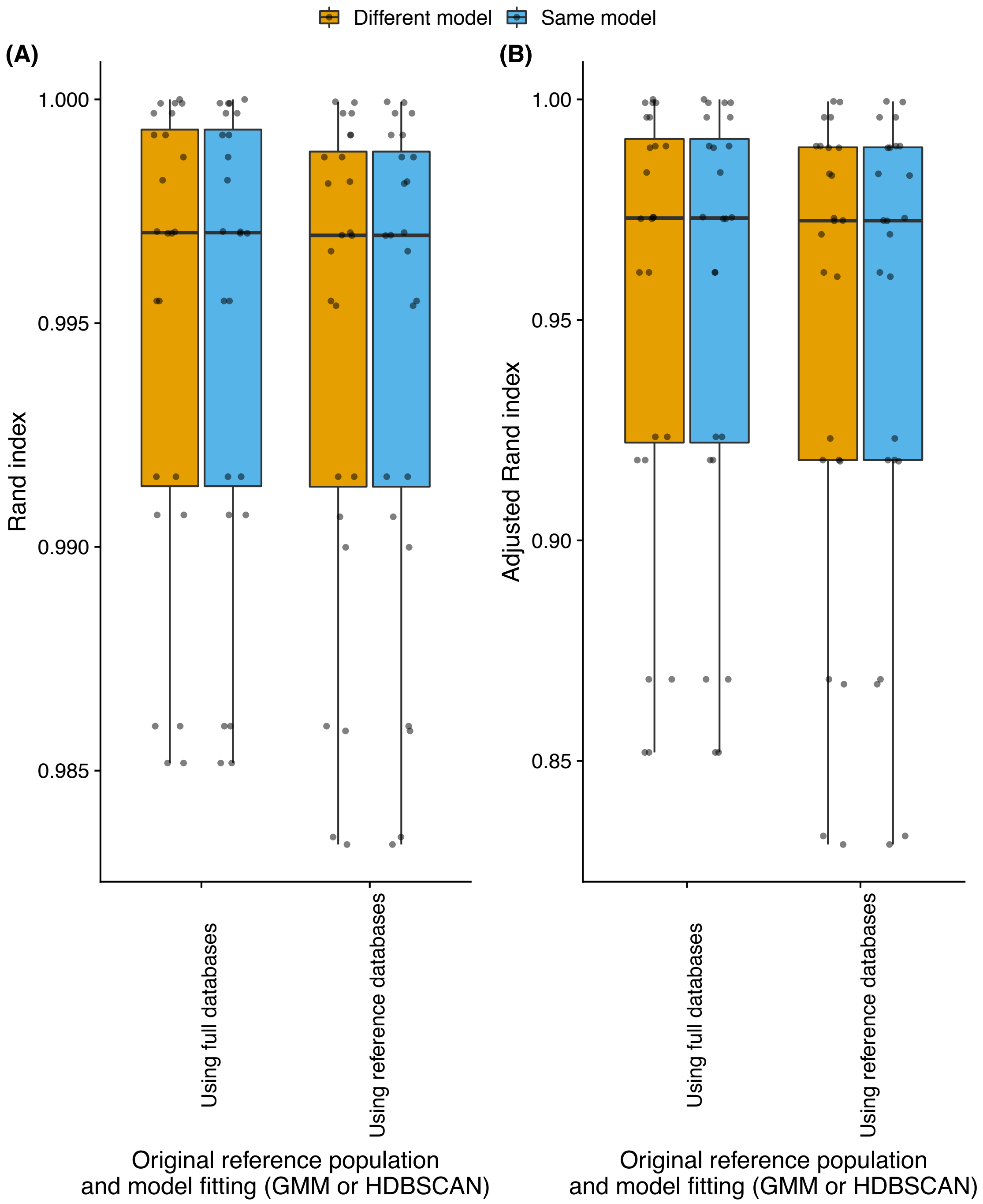

Fig. S15: Comparison of clustering consistency between iterative addition of batches and full clustering of the complete dataset. The set of 4107 draft genomes described in Corander et al. (2017) was clustered by refining both GMM and HDBSCAN models, each fitted to the same set of pairwise distances. These resulted in identical clustering outputs, demonstrating the robustness of PopPUNK's processing to model selection. Each of these clusterings therefore resulted in identical Rand indices when compared to the multiple permutations in which the three non-reference populations were added to the starting Massachusetts or Maela reference database; again, only isolates in the final population to be added were used in this comparison. (A) The Rand indices indicated near-identical clustering when the full database, or a reference-only database, was used to add batches. (B) The adjusted Rand indices for the same comparisons demonstrate the results shown in panel (A) do not represent the chance overlap resulting from the comparison of simple clusterings.

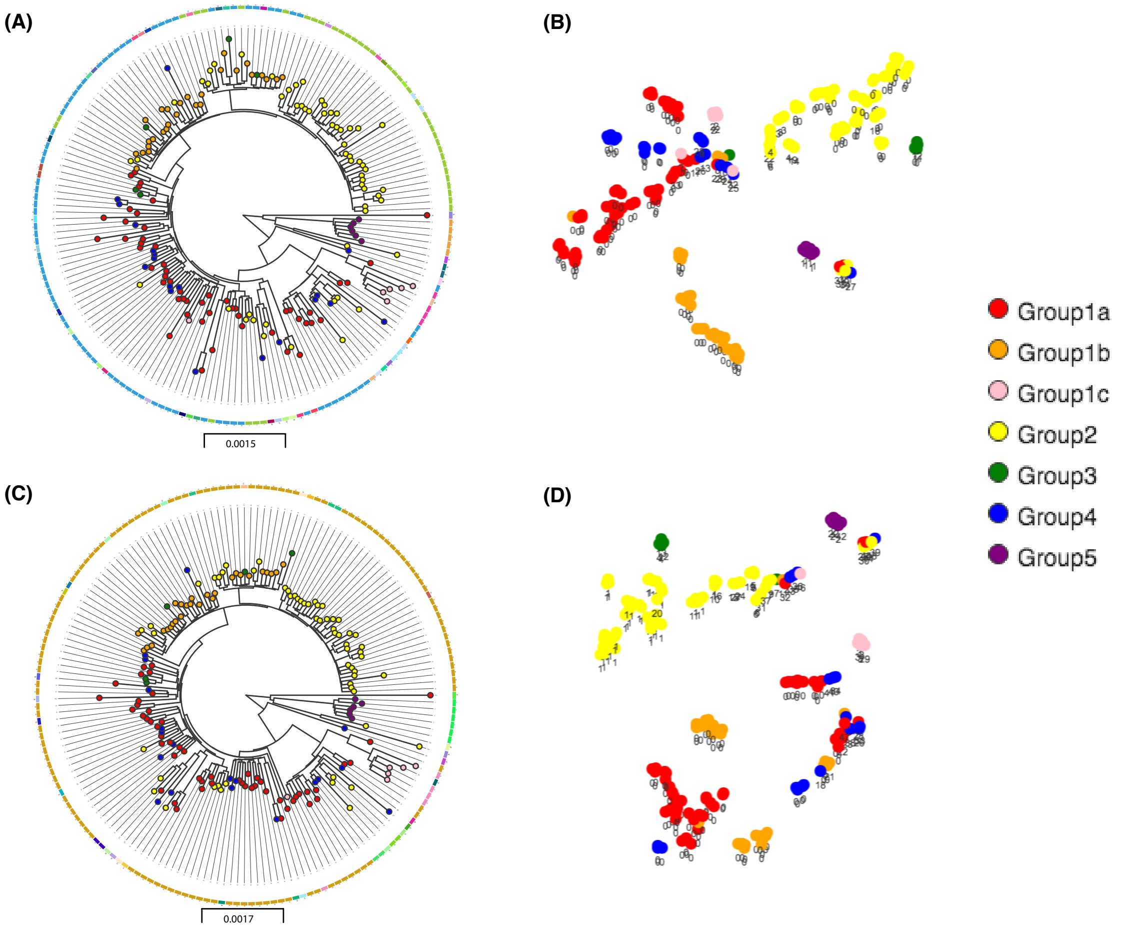

Fig. S16: Comparative analysis of the *S. pneumoniae* PMEN14 population using PopPUNK and PANINI. Using a sketch size of 10^4, (A) a neighbour-joining tree was calculated from the pairwise π distances, and (B) a t-SNE projection was calculated from the pairwise a distances using a perplexity parameter of five. A larger sketch size of 10^5, which affords greater precision when comparing closely-related isolates (Fig S3), was used to calculate (C) a tree and (D) a t-SNE projection in the same manner. The points on the trees and projections corresponding to isolates were coloured according to the accessory genome groupings previously defined using the PANINI output, as indicated by the key. The accessory clusters identified by PopPUNK are indicated by the coloured ring around the phylogeny. These plots demonstrate the robust separation of group one from groups two and three, which were distinguished by the insertion of two prophage, despite their polyphyly (Abudahab et al., 2018). The clade forming group five, which lacks the antibiotic resistance element Tn*916*, was also clearly separated from the rest of the collection.

Supplemental references

Aanensen, D. M. et al. (2016). Whole-Genome Sequencing for Routine Pathogen Surveillance in Public Health: a Population Snapshot of Invasive Staphylococcus aureus in Europe. *MBio* 7 (3).

Abudahab, K., J. M. Prada, Z. Yang, S. D. Bentley, N. J. Croucher, J. Corander, and D. M. Aanensen (2018). PANINI: Pangenome Neighbor Identification for Bacterial Populations. *Microbial Genomics* 4.

Alikhan, N.-F., Z. Zhou, M. J. Sergeant, and M. Achtman (2018). A genomic overview of the population structure of Salmonella. *PLoS Genet.* 14 (4):e1007261.

Bennett, J. S., S. D. Bentley, G. S. Vernikos, M. A. Quail, I. Cherevach, B. White, J. Parkhill, and M. C. J. Maiden (2010). Independent evolution of the core and accessory gene sets in the genus Neisseria: insights gained from the genome of Neisseria lactamica isolate 020-06. *BMC Genomics* 11:652.

Brent, R. P. (1973). *Algorithms for Minimization Without Derivatives*. Courier Corporation.

Cohen, K. A. et al. (2015). Evolution of Extensively Drug-Resistant Tuberculosis over Four Decades: Whole Genome Sequencing and Dating Analysis of Mycobacterium tuberculosis Isolates from KwaZulu-Natal. *PLoS Med.* 12 (9):e1001880.

Corander, J., C. Fraser, M. U. Gutmann, B. Arnold, W. P. Hanage, S. D. Bentley, M. Lipsitch, and N. J. Croucher (2017). Frequency-dependent selection in vaccine-associated pneumococcal population dynamics. *Nature Ecology & Evolution* 1 (12):1950–1960.

Croucher, N. J., C. Chewapreecha, et al. (2014). Evidence for soft selective sweeps in the evolution of pneumococcal multidrug resistance and vaccine escape. *Genome Biol. Evol.* 6 (7):1589–1602.

Croucher, N. J., J. A. Finkelstein, S. I. Pelton, P. K. Mitchell, G. M. Lee, J. Parkhill, S. D. Bentley, W. P. Hanage, and M. Lipsitch (2013). Population genomics of post-vaccine changes in pneumococcal epidemiology. *Nat. Genet.* 45 (6):656–663.

Feng, Y., S. Major, and S. Sievert (2017). rainwoodman/sharedmem 0.3.5.

Grad, Y. H., S. R. Harris, R. D. Kirkcaldy, A. G. Green, D. S. Marks, S. D. Bentley, D. Trees, and M. Lipsitch (2016). Genomic Epidemiology of Gonococcal Resistance to Extended-Spectrum Cephalosporins, Macrolides, and Fluoroquinolones in the United States, 2000-2013. *J. Infect. Dis.* 214 (10):1579–1587.

Hagberg, A. A., D. A. Schult, and P. J. Swart (2008). Exploring Network Structure, Dynamics, and Function using NetworkX. *Proceedings of the 7th Python in Science Conference*. Edited by G. Varoquaux, T. Vaught, and J. Millman. Pasadena, CA USA, p. 11–15.

Hamilton, H. L., N. M. Dominguez, K. J. Schwartz, K. T. Hackett, and J. P. Dillard (2005). Neisseria gonorrhoeae secretes chromosomal DNA via a novel type IV secretion system. *Mol. Microbiol.* 55 (6):1704–1721.

Hubert, L. and P. Arabie (1985). Comparing partitions. *J. Classification* 2 (1):193–218.

Hunter, J. D. (2007). Matplotlib: A 2D graphics environment. *Comput. Sci. Eng.* 9 (3):90–95.

Kallonen, T., H. J. Brodrick, S. R. Harris, J. Corander, N. M. Brown, V. Martin, S. J. Peacock, and J. Parkhill (2017). Systematic longitudinal survey of invasive Escherichia coli in England demonstrates a stable population structure only transiently disturbed by the emergence of ST131. *Genome Res.* 27 (8):1437–1449.

Koelman, D., P. Kremer, J. Lees, M. Brouwer, S. Bentley, and D. van de Beek (2017). Bacterial hypervirulence in Haemophilus influenzae meningitis identified by whole genome sequencing. *J. Neurol. Sci.* 381:181–182.

Kremer, P. H. C. et al. (2017). Benzalkonium tolerance genes and outcome in Listeria monocytogenes meningitis. *Clin. Microbiol. Infect.* 23 (4):265.e1–265.e7.

Lam, S. K., A. Pitrou, and S. Seibert (2015). Numba: A LLVM-based Python JIT Compiler. *Proceedings of the Second Workshop on the LLVM Compiler Infrastructure in HPC*. LLVM '15. New York, NY, USA: ACM, p. 7:1–7:6.

Lees, J. A., M. Galardini, S. D. Bentley, J. N. Weiser, and J. Corander (2018). pyseer: a comprehensive tool for microbial pangenome-wide association studies. *Bioinformatics*.

Lees, J. A., P. H. C. Kremer, et al. (2017). Large scale genomic analysis shows no evidence for pathogen adaptation between the blood and cerebrospinal fluid niches during bacterial meningitis. *Microb Genom* 3 (1):e000103.

Lees, J. A., M. Vehkala, et al. (2016). Sequence element enrichment analysis to determine the genetic basis of bacterial phenotypes. *Nat. Commun.* 7:12797.

McInnes, L., J. Healy, and S. Astels (2017). hdbscan: Hierarchical density based clustering. *The Journal of Open Source Software* 2 (11):205.

Morse, S. A., S. R. Johnson, J. W. Biddle, and M. C. Roberts (1986). High-level tetracycline resistance in Neisseria gonorrhoeae is result of acquisition of streptococcal tetM determinant. *Antimicrob. Agents Chemother.* 30 (5):664–670.

Ondov, B. D., T. J. Treangen, P. Melsted, A. B. Mallonee, N. H. Bergman, S. Koren, and A. M. Phillippy (2016). Mash: fast genome and metagenome distance estimation using MinHash. *Genome Biol.* 17 (1):1–14.

Rand, W. M. (1971). Objective Criteria for the Evaluation of Clustering Methods. *J. Am. Stat. Assoc.* 66 (336):846–850.

Sipola, A., P. Marttinen, and J. Corander (2018). Bacmeta: simulator for genomic evolution in bacterial metapopulations. *Bioinformatics* 34 (13):2308–2310.
